# Counteracting the Ramifications of UVB Irradiation and Photoaging with *Swietenia macrophylla* King Seed

**DOI:** 10.3390/molecules26072000

**Published:** 2021-04-01

**Authors:** Camille Keisha Mahendra, Syafiq Asnawi Zainal Abidin, Thet Thet Htar, Lay-Hong Chuah, Shafi Ullah Khan, Long Chiau Ming, Siah Ying Tang, Priyia Pusparajah, Bey Hing Goh

**Affiliations:** 1 Biofunctional Molecule Exploratory Research Group, School of Pharmacy, Monash University Malaysia, Bandar Sunway 47500, Malaysia; camille.mahendra@monash.edu (C.K.M.); thet.thet.htar@monash.edu (T.T.H.); alice.chuah@monash.edu (L.-H.C.); shafi.khan1@monash.edu (S.U.K.); 2 Liquid Chromatography Mass Spectrometry (LCMS) Platform, Jeffrey Cheah School of Medicine and Health Sciences, Monash University Malaysia, Jalan Lagoon Selatan, Bandar Sunway 47500, Malaysia; syafiq.asnawi@monash.edu; 3 Department of Pharmacy, Abasyn University, Peshawar 25000, Pakistan; 4 PAP Rashidah Sa’adatul Bolkiah Institute of Health Sciences, Universiti Brunei Darussalam, Gadong BE1410, Brunei; longchiauming@gmail.com; 5 Chemical Engineering Discipline, School of Engineering, Monash University Malaysia, Bandar Sunway 47500, Malaysia; patrick.tang@monash.edu; 6 Advanced Engineering Platform, School of Engineering, Monash University Malaysia, Bandar Sunway 47500, Malaysia; 7 Tropical Medicine and Biology Platform, School of Science, Monash University Malaysia, Bandar Sunway 47500, Malaysia; 8 Medical Health and Translational Research Group, Jeffrey Cheah School of Medicine and Health Sciences, Monash University Malaysia, Bandar Sunway 47500, Malaysia; 9 College of Pharmaceutical Sciences, Zhejiang University, 866 Yuhangtang Road, Hangzhou 310058, China; 10 Health and Well-Being Cluster, Global Asia in the 21st Century (GA21) Platform, Monash University Malaysia, Bandar Sunway 47500, Malaysia

**Keywords:** photoaging, proteomics, genomics, *Swietenia macrophylla*, UV irradiation, keratinocytes, epidermal layer, cosmetics, natural product, LC-MS/MS

## Abstract

In this day and age, the expectation of cosmetic products to effectively slow down skin photoaging is constantly increasing. However, the detrimental effects of UVB on the skin are not easy to tackle as UVB dysregulates a wide range of molecular changes on the cellular level. In our research, irradiated keratinocyte cells not only experienced a compromise in their redox system, but processes from RNA translation to protein synthesis and folding were also affected. Aside from this, proteins involved in various other processes like DNA repair and maintenance, glycolysis, cell growth, proliferation, and migration were affected while the cells approached imminent cell death. Additionally, the collagen degradation pathway was also activated by UVB irradiation through the upregulation of inflammatory and collagen degrading markers. Nevertheless, with the treatment of *Swietenia macrophylla* (*S. macrophylla*) seed extract and fractions, the dysregulation of many genes and proteins by UVB was reversed. The reversal effects were particularly promising with the *S. macrophylla* hexane fraction (SMHF) and *S. macrophylla* ethyl acetate fraction (SMEAF). SMHF was able to oppose the detrimental effects of UVB in several different processes such as the redox system, DNA repair and maintenance, RNA transcription to translation, protein maintenance and synthesis, cell growth, migration and proliferation, and cell glycolysis, while SMEAF successfully suppressed markers related to skin inflammation, collagen degradation, and cell apoptosis. Thus, in summary, our research not only provided a deeper insight into the molecular changes within irradiated keratinocytes, but also serves as a model platform for future cosmetic research to build upon. Subsequently, both SMHF and SMEAF also displayed potential photoprotective properties that warrant further fractionation and in vivo clinical trials to investigate and obtain potential novel bioactive compounds against photoaging.

## 1. Introduction

The existence of ultraviolet radiation (UVR) in our lives is very much like a double-edged sword. On one hand, we cannot live without it, however, excessive exposure could also lead to our demise. This is especially true for UVB, the reasons being that through it, our bodies produce the much-needed vitamin D, however, conversely, among the two types of UVR in our atmosphere, it is the one that causes the most harm to our skin, with photodamage ranging from sunburns to skin carcinogenesis [1]. Another benefit of UVB, or more precisely narrowband UVB with an emission peak of 311 nm, is its wide use in phototherapy treatments against skin diseases such as psoriasis, mycosis fungoides, vitiligo, etc. It has been witnessed that UVB phototherapy often improved skin conditions of diseased skin, giving temporary periods of respite to the patients [2,3,4]. Nevertheless, regardless of its benefits, overexposure to UVB is more often than not the case for the general population. This is often showcased in the appearance of irregular pigmentation, fine lines, wrinkles, poor texture, sagging skin, etc., on our skin after prolonged exposure, which are key signs of skin photoaging [5]. Although there are ‘intrinsic’ factors like the natural generation of reactive oxygen species (ROS) and reactive nitrogen species (RNS) in our skin and ‘extrinsic’ factors such as lifestyles changes and environmental pollution that causes skin aging, it is still undeniable that the exposure of UVB plays a part in skin aging [6,7,8].

There are three categories of UVR based on their wavelengths: UVA, UVB, and UVC. Among the three kinds of UVR, only UVA and UVB can penetrate through the ozone, with UVA (320–400 nm) having a 95% penetration level and UVB (290–320 nm) with a maximum penetration level of 5% through the ozone layer [9]. Despite the wavelength of UVB being much shorter than UVA and therefore, mostly absorbed through the epidermal layer, its detrimental effect is not limited to the epidermal layer [10]. Direct penetration of UVB not only form cyclobutene pyrimidine dimers (CPD) and pyrimidine-pyrimidone (6–4) photoproducts in the DNA, but also incites the production of ROS and RNS in the skin [11,12,13]. This then increases the oxidative stress levels in the skin, which quickly depletes the skin’s antioxidant defense, and initiates a cascade of pro-inflammatory and other intracellular signals like matrix metalloproteases (MMP) and melanogenic cytokines by keratinocyte cells [14,15,16]. Ultimately, this leads to the formation of unwanted irregular pigmentation through the activation of the tyrosinase family, and the degradation of our skin’s extracellular matrixes, reducing the elasticity of the skin and forming wrinkles [15,16,17]. Thus, in this study, the impact of UVB on keratinocytes was investigated. The purpose of this was to not only produce a wider view, and therefore better understanding of the molecular changes and pathway influenced by UVB, but also to aid in the advancement of cosmetic products that can better counteract the photodamaging effects of UVB. To achieve this, the Nanoflow-Ultra High-Performance Liquid Chromatography-Tandem Mass Spectrometry (LC-MS/MS) platform was utilized in the proteomics analysis of this study. The LC-MS/MS is a high throughput platform that is capable of accurately measuring fold changes and identifying a large range of proteins and peptides with the aid of bioinformatics in a relatively short time while remaining cost-effective. Another benefit of LC-MS/MS is its ability to separate and distinguish structurally or chemically similar peptides and proteins from each other [18].

Furthermore, this research also evaluated the capabilities of *S. macrophylla* seed extract as a photoprotective agent. *S. macrophylla* is a timber tree from the Meliaceae family that can be found in the tropics of Central America, Southeast Asia, and Mexico [19,20,21]. Besides being well prized for its mahogany wood, its seeds, containing flavonoids, alkaloids, and saponins, are often used in traditional medicine to treat sicknesses such as diabetes, hypertension, and even physical pain [22,23]. To prove its medicinal claim, many studies had been conducted, and through them, it has been reported that the seed possesses anti-cancer, neuroprotection, anti-hyperglycemic, anti-inflammation, antioxidant, and anti-viral properties [21,23,24,25,26,27,28]. Recently, it was discovered that one of the limonoid compounds, swietenine, isolated from the seed were responsible for the seed’s antioxidant and anti-inflammatory activity on LPSEc stimulated RAW264.7 murine macrophage. Not only was the compound able to significantly inhibit the production of nitric oxide, but it also engaged the nuclear factor erythroid 2 (NRF2)/heme oxygenase-1 (HO-1) antioxidant pathway while downregulating the production of pro-inflammatory markers like interleukin (IL)-1β, tumor necrosis factor (TNF)-α, interferon gamma (IFN-γ), IL-6, cyclooxygenase (COX-2), and nuclear factor-κB (NF-κB) [28]. On the other hand, its wound healing ability has also been evaluated by Nilugal et al. [29]. In their study, the application of *S. macrophylla* ethanolic seed extract ointment was seen to significantly speed up the healing process of the excised wounds on the rats [29]. Thus, based on these claims, especially those regarding its antioxidant, wound healing, and anti-inflammatory properties, it would prove interesting to investigate if the seed extract and fractions can act as a photoprotective reagent against UVB and therefore be a potential active ingredient in the formulation of photoprotective cosmetics given the reasons that those aforementioned properties are inherently important in counteracting UVB-induced photodamage.

## 2. Results and Discussion

### 2.1. Cytotoxicity Assessment of S. macrophylla Extract and Fractions

HaCaT cells were treated with various concentrations (0–100 µg/mL) of the extract and fractions for 24 h. According to the data obtained, *S. macrophylla* crude extract (SMCE) begins to induce a dose-dependent decrease in cell viability starting from the concentration of 12.5 μg/mL with cell viability of 87.5 ± 3% (*p* ≤ 0.01). The cell viability then continues to decrease to 74.83 ± 4.94% (*p* ≤ 0.001), 51.77 ± 3.96% (*p* ≤ 0.001), and 44.36 ± 3.36% (*p* ≤ 0.001) when treated with 25, 50, and 100 μg/mL SMCE, respectively. On the other hand, after fractionation, SMHF did not induce any significant decrease in cell viability, even at concentrations as high as 100 μg/mL. As for SMEAF, cell viability was significantly decreased dose-dependently instead at concentrations of 25, 50, and 100 μg/mL to 82.04 ± 5.4% (*p* ≤ 0.001), 49.93 ± 3.63% (*p* ≤ 0.001), 35.25 ± 7.76% (*p* ≤ 0.001), respectively. Finally, *S. macrophylla* water fraction (SMWF) became cytotoxic toward HaCaT at 100 μg/mL with cell viability of 80.7 ± 6.15% (*p* ≤ 0.001) compared to the untreated control cells. Among the four samples, SMCE had the highest cytotoxicity against HaCaT, but the cytotoxicity levels were reduced after fractionation. This demonstrates that synergistic cytotoxic compounds were most likely separated during fractionation, and therefore, the fractions had reduced cytotoxic levels compared to the crude extract itself. Following the data obtained, the concentration 6.25 μg/mL for SMCE, 100 μg/mL for SMHF, 12.5 μg/mL for SMEAF, and 50 μg/mL for SMWF were chosen as the non-cytotoxic concentration to treat the cells in the upcoming experiments.

### 2.2. The Dynamic Proteomics and Genomic Dysregulation in Keratinocyte Cells Effectuated by UVB and Its Attenuation by S. macrophylla

#### 2.2.1. Analysis of UVB-Induced Protein Modifications and the Reversal Effect of *S. macrophylla*

**Figure 1:**
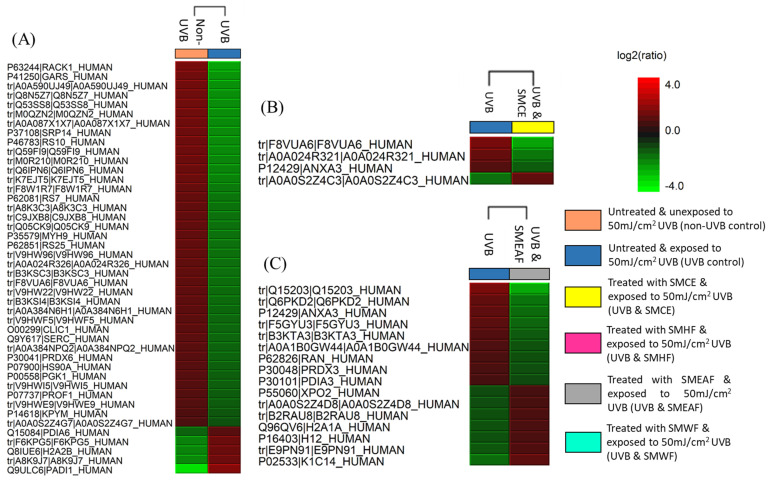

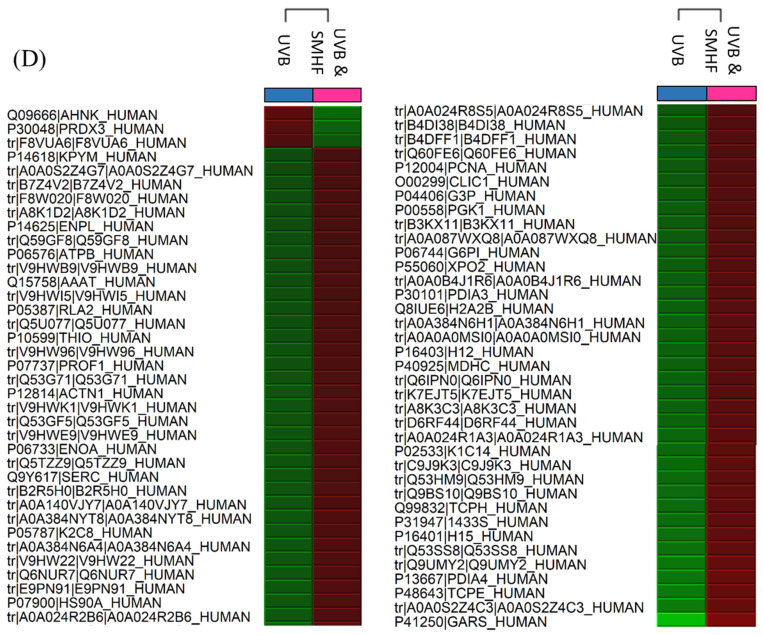

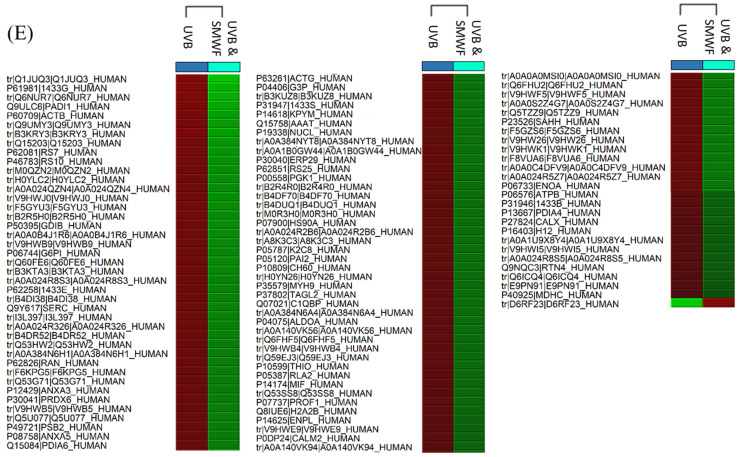
Heat maps obtained from Peaks Q displaying the changes in protein expression across controls and samples. The red color signifies the upregulation of protein expression while the green color signifies downregulation of protein expression. (**A**) The comparison of non-UVB and UVB control cells. (**B**–**E**) are the comparison of UVB control cells with cells that were simultaneously exposed to UVB while being treated with SMCE, SMEAF, SMHF, and SMWF, respectively.

**Figure 2:**
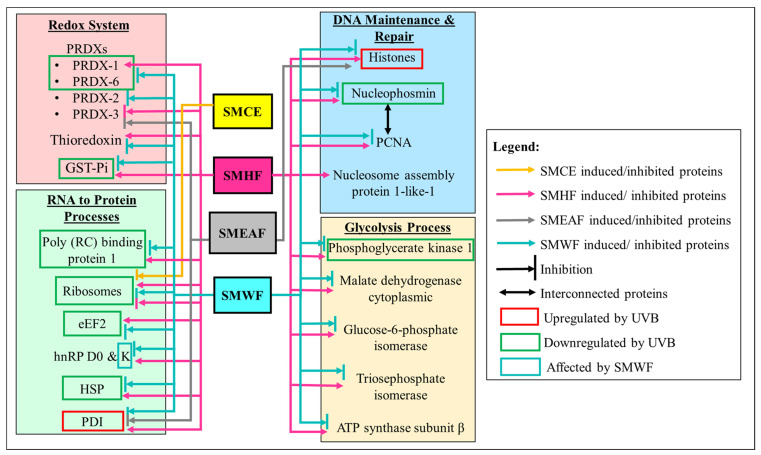
Dysregulation of proteins involved in the redox system, DNA maintenance and repair, RNA transcription to protein synthesis, and glycolysis process by UVB irradiation and the effect SMCE, SMHF, SMEAF, and SMWF has on the UVB irradiated cells.

**Figure 3:**
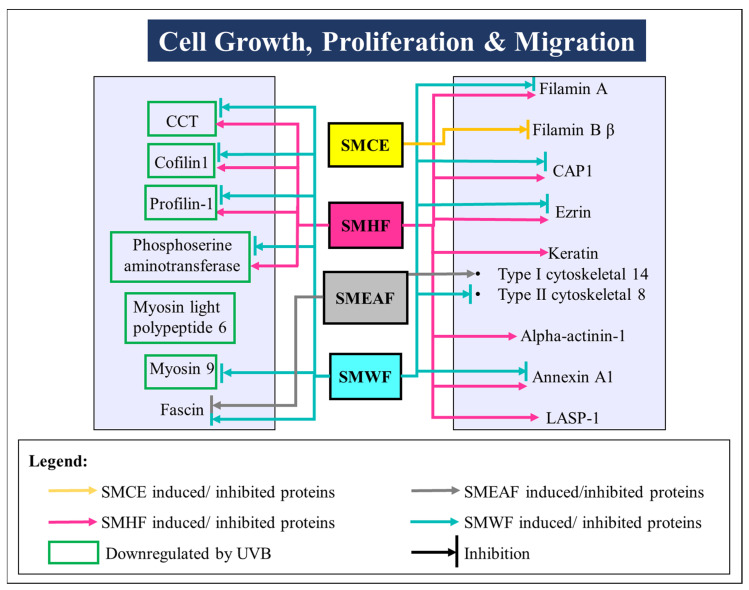
UVB-induced changes in the expression of proteins involved in cell growth, proliferation, and migration processes. These proteins then were either rescued or further suppressed after being treated with SMCE, SMHF, SMEAF, and SMWF.

To ascertain the effect of UVB irradiation, with or without the treatment of *S. macrophylla*, on the protein expression changes in HaCaT cells, a high throughput proteomics analysis using LC-MS/MS was conducted. For the controls, untreated HaCaT cells were either unexposed or exposed with 50 mJ/cm^2^ UVB to obtain the unexposed (non-UVB) and exposed (UVB) controls, respectively. Protein samples were then collected at the 24 h time point. Similarly, cells treated with *S. macrophylla* extract and fractions were also irradiated with 50 mJ/cm^2^ UVB and their protein samples were individually collected at the 24 h time point. After processing and identifying individual proteins with the Uniprot database, a comparison of protein expression (in ratio) was obtained via PEAKS Q, based on the area under the curve. Five heatmaps (Figure 1A–E) depicting the comparison between the two untreated (non-UVB and UVB) controls and the difference between the UVB control and *S. macrophylla* treated cells were also obtained from PEAKS Q. Next, to truly compare amongst the data obtained, a compiled list of significant (*p* ≤ 0.05) differentially expressed proteins, from the controls and *S. macrophylla* treated cells, were drawn up in Table 1. Additional detailed data of each protein can subsequently be found in Supplementary Materials Table S1. In Table 1, proteins obtained from both non-UVB and UVB controls were first compared to determine the protein expression changes induced by UVB. This is then followed by the changes seen in the treated cells compared to the UVB control. Proteins of similar Uniprot ID or name were directly compared across all groups for a clear comparison. When there are no significant changes seen in the protein ratio between the two comparing groups, the label N/S (not significant) will be assigned. In total, 151 proteins were identified to be differentially expressed among the controls and treated cells. From these differentially expressed proteins, it can be seen that UVB exposure has detrimental effects on a wide range of molecular functions such as DNA maintenance and repair, RNA synthesis, protein synthesis and processing (biogenesis, folding, stabilizing, proteostasis, etc.), cell growth, glycolysis process, etc., which ultimately determines the survival of the cells. When treated with SMCE, only four proteins within the HaCaT cells (60 S ribosomal protein L18, fumarate hydratase, annexin A3, and filamin B β) showed significant changes in expression in comparison to the UVB control. Other than fumarate hydratase, the three other proteins were instead significantly decreased in their expression levels. In contrast, SMHF showed the most attenuation in the differentially expressed proteins compared to the UVB control. Of all the proteins, only three proteins, 60 S ribosomal protein L18, neuroblast differentiation-associated protein AHNAK (AHNAK), and peroxiredoxin (PRDX)-3, were downregulated by SMHF. Aside from that, SMEAF induced changes in only 16 proteins in which seven of them were upregulated and nine were downregulated in comparison to the UVB control. The proteins that were upregulated were histone H2A type 1-A, histone H1.2, keratin type I cytoskeletal 14, exportin-2, nucleolar and coiled-body phosphoprotein 1 (NOLC1), and protein kinase C substrate 80 K–H isoform. Vice versa, PRDX-3, protein disulfide-isomerase (PDI) A3, annexin A3, polyubiquitin-C, HNRPCL1 protein, fascin, cathepsin D, prothymosin alpha, and GTP-binding nuclear protein Ran were downregulated by SMEAF. Finally, SMWF displayed significant downregulation of all proteins except the receptor of activated protein C kinase 1 (RACK-1) was upregulated. To elucidate the impact of *S. macrophylla* extract and fractions on UVB irradiated HaCaT cells, the functions of each protein were further studied and classified accordingly. An overview of the proteins affected by UVB and treatment is depicted in Figure 2 and Figure 3 based on the data obtained. Figure 2 depicts the changes in protein expression occurring in various systems such as the redox system, DNA maintenance and repair, RNA transcription to protein processing, and the glycolysis process. On the other hand, Figure 3 covers proteins involved in cell growth, proliferation, and migration.

#### 2.2.2. Oxidative Damage Induced Activation of the Redox System

As UVB irradiation induces oxidative damage, it is expected that there would be changes in the molecular dynamics within the keratinocyte cells and one of them is the redox regulating PRDXs. PRDXs are a family of antioxidant enzymes with an aptitude for reducing alkyl hydroperoxide and hydrogen peroxide (H_2_O_2_) to alcohol and water, respectively [30]. In mammals, six distinct PRDXs have been discovered and can be found mostly in the cytosol, but some can also be found in the nucleus, mitochondria, lysosome, endoplasmic reticulum, or be secreted from the cell [31,32]. The secretion of PRDX like PRDX-2 triggers the production and secretion of TNF-α in macrophages, inducing a cascade of inflammatory response to the stimuli [33]. PRDXs also work together with thioredoxin and thioredoxin reductase in its redox reaction by obtaining electrons from thioredoxin/thioredoxin reductase systems [31]. It is through this relationship that H_2_O_2_ cell signaling is mediated and controlled, even in the event of PRDXs hyperoxidation [34,35]. The changes in PRDXs by UVB was previously reported in various studies on skin cells. In a study done by Liu et al. [36], PRDX 1 was upregulated after exposing HaCaT cells to 21 ± 1 mJ/cm^2^ UVB for 18 h and then incubated for another 6 h before protein extraction. Another study by Wu et al. [37] on skin fibroblasts displayed that at low (17 mJ/cm^2^) and middle (70 mJ/cm^2^) doses of UVB irradiation, the human fibroblast cells exhibited an increase in PRDX-1, but for PRDX-4 and -6, this increase was only seen in the middle dose of UVB irradiation at the 24 h time point. At low doses of UVB, the PRDX-4 and -6 were negatively regulated as are all PRDXs at high (468 mJ/cm^2^) dose of UVB. Although different results on PRDX expression levels were obtained due to different exposure levels, protein harvest times, and cell type, it is undeniable that UVB does affect the expression of PRDX. Knockdown of PRDX-3 and mutations in PRDX-6 in mice even showed increased keratinocyte apoptosis and tissue damage, respectively, due to increased susceptibility in oxidative damage [30,38], thus, indicating the importance of PRDX in cell survival against UVB damage.

In response to UVB irradiation, PRDX-1 and -6 were significantly downregulated but there were no significant changes in the expression of PRDX-2 and-3 as can be seen in Table 1 and Figure 2. No changes in any PRDX levels in comparison to the UVB control cells were observed after treating with SMCE. Despite that, its fraction, SMHF, initiated an upregulation in PRDX-1 and, subsequently, downregulated PRDX-3. No changes in PRDX-6 levels were detected, though, when the cells were treated with SMHF. Furthermore, SMHF also upregulated the expression of thioredoxin, suggesting that SMHF might have possibly activated the thioredoxin/thioredoxin reductase system along with PRDX-1. On another note, SMEAF also induced similar downregulation of PRDX-3 in the HaCaT cells as implicated by SMHF, but incited no changes in other PRDXs. As for SMWF, downregulation of all PRDX-1, -2, and, -6 were recorded.

The increase in PRDX-1 and thioredoxin in cells treated with SMHF suggests that the compounds in SMHF are not only able to counteract against UVB induced H_2_O_2_, but might also indirectly inhibit the activation of the apoptosis signal-regulating kinase-1 (ASK-1)-mediated apoptotic pathway. According to Kim et al. [39], PRDX-1 is able to negatively regulate ASK-1, a mitogen activated protein (MAP) kinase kinase, activity and it is through the inhibition of ASK-1 activation that PRDX-1 is able to inhibit MAP kinase kinase (MKK)3/6, Jun N-terminal kinase (JNK), and p38 phosphorylation, ultimately attenuating cell apoptosis. Another interesting point to note is that the expression of GST-pi, which was previously downregulated in UVB control cells compared to the non-UVB control cells, were now upregulated under the treatment of SMHF. This change in expression of GST-pi is intriguing because GST-pi is a known inhibitor of JNK. Under normal cell conditions, monomeric GST-pi forms a GST-pi-JNK complex with c-Jun, actively inhibiting the phosphorylation of JNK. However, under oxidative stress, monomeric GST-pi becomes detached from the complex and forms dimerization or multimerization of GST-pi. This enables JNK and c-Jun to be activated. Nevertheless, it is believed that under conditions where newly synthesized GST-pi is formed, the GST-pi-JNK complex will once again reform, effectively inhibiting the activation of JNK [40]. In short, with the increase in expression of PRDX-1, thioredoxin, and GST-pi protein, it is possible that SMHF might be able to improve cell survival in UVB irradiated keratinocytes. Nevertheless, this hypothesis needs to be investigated further as SMHF also suppresses PRDX-3, which also plays an important role as an antioxidant enzyme against cell apoptosis. As for SMCE, SMEAF, and SMHF, it can be seen that neither of these extracts and fractions can inhibit cell apoptosis via PRDXs.

#### 2.2.3. Impact of UVB on DNA Maintenance and Repair in Keratinocyte Cells

Histones are indispensable in the regulation of all nuclear processes such as DNA replication, the progression of cell- cycle, transcription, etc. Assembled with two copies of histone H2A, H2B, H3, and H4 histone each, these histones form the nucleosome core particle, which wraps around 146 bp of DNA. To further stabilize the octameric core, H1 linker histone comes into play to form chromatin-specific high-order structures. Other additional histone variants and accessory proteins are also distributed along the chromatin fiber [41]. Due to the importance of its role in epigenetic regulation, any changes in histone modification or expression level will impact gene expression and determine cell fate. Based on the analysis, this study showed that the exposure of HaCaT cells to UVB upregulated the expression of histone H2A type 2-B and H2B (Table 1). This upregulation of histones is not uncommon under the phototoxicity of UVB. Both Sesto et al. [42] and Dazard et al. [43] had previously reported that UVB irradiation causes the expression of histones to be upregulated at the 24th hour in human keratinocytes after exposure. Dazard, Gal, Amariglio, Rechavi, Domany, and Givol [43] even suggested that the upregulation of histones could aid in DNA repair, but as of yet, the process has not been fully confirmed.

When the cells were treated with SMCE, there were no significant changes in expression level. However, after fractionation, SMHF not only further increased the expression of histone H2A type 1-B, but also H1.2 and H1.5. SMEAF also upregulated histone H1.2 and H2A type 1-A. In contrast, SMWF was the only fraction that downregulated histone H2A type 2-B, H2B, H1.2, and H4. Although, from this data it is possible that SMHF and SMEAF might be implicated in processes involving DNA repair and maintenance, however, the increased expression of H1.2 by both extracts is concerning. This is because, when histone H1.2 translocates from the nucleus to the cytosol, it has been reported to become pro-apoptotic and is able to induce cell death by interacting with the cytochrome C and proapoptotic Bcl-2 family in x-ray irradiated rat cells [44]. This is further supported by Ruiz-Vela and Korsmeyer [45], who showed that histone H1.2 promotes the activation of caspase 3 and 7 via apoptotic protease activating factor 1 (APAF-1) and caspase-9 in the UV induced apoptosis process. Hence, further studies must be done to better elucidate the effect SMHF and SMEAF have on histones.

On the other hand, nucleophosmin, a nucleolar phosphoprotein, plays an essential role in cell proliferation, ribosome biogenesis, and cell survival in DNA-damaged cells [46]. Some of the existing findings on the effect of UV on nucleophosmin showed that UV exposure upregulates nucleophosmin dose-dependently and the increase in nucleophosmin reduces cell death [47,48]. Similar observations on nucleophosmin upregulation were also seen when fibroblast cells were exposed separately to either UVA or UVB [37,49]. Subsequently, overexpression of nucleophosmin suppressed both p53 and p21 expression, although the limit of suppression on p21 stops at a certain dose of UV radiation. Higher doses of UV are unable to inhibit p21 expression, signifying that nucleophosmin may function as an early sensor mechanism against genotoxic stress [47]. The lack of nucleophosmin also arrested the cell cycle in the G2 phase following UV exposure, resulting in a delay of cell mitosis and proliferation [47]. Another study showed that nucleophosmin is able to increase PCNA expression by regulating the PCNA promoter, thus mediating DNA repair via the nucleotide excision repair pathway [48]. However, in this study, nucleophosmin was downregulated instead under UVB phototoxicity as can be seen in Figure 2. This suggests that the process of DNA repair had been inhibited by UVB at 24 h after exposure, thus encouraging the cells toward cell death. Although SMCE does not affect the expression levels of nucleophosmin, SMHF was able to inverse the effect UVB had on nucleophosmin. Furthermore, an increase in PCNA by SMHF was also observed, further emphasizing the potential of SMHF in initiating DNA repair in UV-damaged cells. Additionally, although these proteins had no changes between the UVB and non-UVB control, upregulation of these two proteins by SMHF might indirectly affect the DNA repair mechanism and even cell survival in keratinocyte cells. One of the proteins that were upregulated is the nucleosome assembly protein 1-like 1. This protein is known for its role in histones H2A and H2B transportation into the nucleus and also its ability to remove and replace H2A-H2B or other histone variant dimers, facilitating nucleosome sliding along the DNA in favor of thermodynamically better positions [50,51]. Another is the protein, GAPDH. Traditionally, this protein is commonly used as a housekeeping gene, but this protein is in fact involved in many different cellular processes such as DNA repair, membrane fusion and transport, tRNA export, cytoskeletal dynamics, and even cell death [52,53,54,55,56,57]. Therefore, more studies on SMHF are warranted. On the flip side, SMWF lowered the expression of nucleophosmin, PCNA, and GAPDH, implicating its inhibitory effect on DNA maintenance and repair in UVB damaged cells. Subsequently, SMEAF itself did not affect any of these proteins.

#### 2.2.4. Modifications of Downstream Process from RNA to Protein

In addition to DNA damage, poly (RC) binding protein 1, which is responsible for RNA transcription, splicing, and translation were also downregulated by UVB exposure, affecting downstream processes of protein synthesis. Other proteins involved in protein synthesis are also dysregulated by UVB as depicted in Figure 2. These proteins are ribosomes and eEF2, a protein that acts as a catalyst to the ribosomes in shifting the mRNA-tRNA complex within ribosomes from the 5′ end to 3′ end in a process called translocation [58]. Apart from being involved in protein synthesis, ribosomes like ribosomal protein L12 are also involved in the translocation of newly synthesized ribosome protein from the cytoplasm to the nucleus with importin 11 [59]. Under the exposure of 100 mJ/cm^2^ UVB, the ribosomal protein gene expression in primary human keratinocyte cells was reported to be significantly increased 6 h after exposure [60]. However, this increase in expression was significantly reduced at 24 h, along with the expression of eEF2. Following the treatment of cells with *S. macrophylla* extract and fractions, SMCE was seen to further reduce the expression of 60S ribosomal protein L18, but did not change the expression level of poly (RC) binding protein 1 or eEF2. Nonetheless, SMHF significantly elevated poly (RC) binding protein 1, several ribosomal proteins, and eEF2 levels. SMHF also increased the expression of hnRP D0 and K, in which both are of the same hnRP family as poly (RC) binding protein 1 and are also involved in RNA regulation either through DNA or RNA binding [61,62,63]. Again, SMEAF induced no changes in the expression of the proteins mentioned, while a completely opposite effect was obtained from cells treated with SMWF. The expression of various ribosomes, poly (RC) binding protein 1, eEF2, and hnRP K was diminished under the treatment of SMWF. Hence, it can be suggested that only SMHF might be able to augment the protein synthesis pathway after UVB.

On another note, under environmental stresses such as UVB exposure, the overexpression of ROS depletes Ca^2+^ ions from the endoplasmic reticulum (ER) lumen. Subsequently, this leads to the malfunction of ER chaperones and other proteins, which then leads to the accumulation of unfolded or misfolded proteins, promoting ER stress [64]. In the event this occurs, HSPs are expressed. HSPs are proteins that are expressed in response to cellular stress, for example, oxidative damage, chemical stress, hyperthermia, etc., and are commonly known for their maintenance of cellular proteostasis [65,66]. However, their location and physiological role vary according to their classification, which can be separated based on their molecular size that ranges from 10 to more than 100 kDa [67]. In addition, the expression of HSP has been correlated to cell survival [68]. As reported by Merwald et al. [69], increased expression of HSP-72 through prior induction demonstrated increased keratinocyte cell survival under UVB irradiation in comparison to the control. This suggests that the expression of HSP is key to stress tolerance and the survival of keratinocytes against phototoxicity.

The expression level of HSPs has been noted to change according to various time points after UVB exposure. In a study where the cellular protein was collected at the 5 h time point, HSP-60 and -70 kDa was increased in normal human epithelial keratinocyte cells [70]. However, at the 12th hour, Howell et al. [71] reported a decrease in the mRNA of heat shock cognate 71 kDa protein. Consecutively, in this study, the expression of two HSPs, HSP-70 kDa protein 8 and HSP-90α was significantly downregulated compared to the non-UVB control 24 h after exposure to 50 mJ/cm^2^ UVB. It is possible that after the initial exposure of UVB, the expression of HSP is triggered and thus increases rapidly, but then the expression will slowly decrease with time as cell death occurs. Nevertheless, with the treatment of SMHF, not only was the expression of the two HSPs upregulated, but three other HSP, HSP-10, HSP-70 kDa protein 4, and HSP-70 kDa protein 9, were also upregulated. In contrast, SMWF was seen to significantly further downregulate the expression of various HSPs while no changes in HSP expression were seen in both SMCE and SMEAF.

The upregulation of HSP-10, HSP-70, and HSP-90 kDa by SMHF as seen in Table 1 is a good sign of cell survival as their functions are important in cell maintenance. HSP-10 kDa, in conjuncture with HSP-60, was reported to inhibit cell apoptosis by modulating the Bcl-2 family and thereby the intrinsic apoptotic pathway in cardiac muscle cells [72]. On the other hand, HSP-70 kDa is involved in the folding, assembly, and refolding of aggregated and misfolded proteins. They also control the membrane translocation of proteins and regulate the activity of regulatory proteins [73]. Finally, HSP-90 kDa acts as an actin-binding protein, modulating cell migration, which is essential for wound healing [74,75]. Topical application of HSP90α infused in 10% carboxymethylcellulose cream on a 1 × 1 cm wound located on the back of nude mice for five days significantly accelerated wound closure. Further analysis of the mice skin revealed that the HSP90α cream instigated significant re-epithelialization and formed thicker and longer epidermis compared to the control [76]. Hence, the increase of these HSPs by SMHF is a good indicator of its potential photoprotective and wound healing abilities.

Besides HSPs, the presence of PDI under oxidative stress is also essential to cell survival. PDI plays an important role in adding disulfide bonds into proteins through the oxidase activity and via the isomerase activity, which aids in the rearrangement of incorrect disulfide bonds [77]. This means that through PDI, there are fewer misfolded proteins as disulfide bonds are vital in maintaining the structure, regulation, and function of proteins [78]. Although PDIs are anti-apoptotic biomarkers for tumor growth, metastasis, and angiogenesis, it may yet be an essential protein to look into and upregulate to promote cell survival and proliferation in skin cells after UVB irradiation [79,80]. In human fibroblast cells, both PDI and PDI-A3 were significantly upregulated 24 h after being exposed to 70 mJ/cm^2^ UVB dosage [37]. Another study on the 3D cell culture of CDD 1102 KERTr human keratinocyte cell line also displayed a significant increase in PDI protein expression 24 h after exposure to 60 mJ/cm^2^ UVB [81]. Similarly, in this study, PDI-A6 protein levels were significantly increased after UVB exposure while no changes in the other PDIs were seen (Table 1 and Figure 2). Treatment with SMCE induced no difference in the expression of PDI. Nevertheless, with the treatment of SMHF, not only were the increased levels of PDI-A6 maintained, but the PDI-A3 and PDI-A4 levels were also significantly elevated when compared to the UVB control. The increase in PDIs after UVB irradiation by SMHF is a good indication of the treatment in combat against UVB phototoxicity. On the other hand, SMEAF suppressed the expression of PDI-A3 while SMWF downregulated PDI-A4 and PDI-A6.

#### 2.2.5. UVB Exposure Affects Cell Growth, Proliferation, and Migration

The dysregulation of actin cytoskeleton dynamics via UVB exposure could bring about the disruption of cell growth, division, migration, and proliferation necessary for wound healing, leading to cell death. In this experiment, proteins such as chaperonin-containing t-complex polypeptide 1 (CCT) -β and δ, cofilin 1, profilin 1, phosphoserine aminotransferase, myosin light polypeptide 6, and myosin 9 experienced significant downregulation 24 h after the exposure of UVB on HaCaT. These proteins are, in one way or another, connected to the regulation of actin rearrangement and cytoskeleton organization. For example, CCT, a cytosolic molecular chaperone, is known to assist in the folding of tubulin, actin, and many other cytosolic proteins [82]. It also acts as a modulator in the process of assembling the cell cytoskeleton [83]. Cofilin1 and profilin-1, on the other hand, work hand in hand with CAP1 and various other actin-associated proteins in actin filamin turn over [84,85]. Aberrant expressions of these proteins will result in the alteration of cell–cell adhesion, cell proliferation, and motility [85,86,87]. Next, phosphoserine aminotransferase, an enzyme involved in serine biosynthesis, has also been implicated in an increase in the proliferation of cells [88]. It was reported that when phosphoserine aminotransferase was suppressed, alterations in cell morphology and F-actin cytoskeletal arrangement was seen. Furthermore, the suppression also inhibited the migration and motility of triple negative breast cancer [89]. Finally, myosins are actin-based molecular motors that function to bind actin filaments together [90]. When the skin is wounded, myosin II, made partly of myosin 9, generates contractile forces necessary for cell motility and migration into the wounded area [91].

When the cells were treated with *S. macrophylla* extract and fractions, SMCE can be seen to decrease the expression of filamin B β. Filamins are actin-binding proteins that act as crosslinks to the actin cytoskeleton filaments to form a dynamic structure. They also aid in anchoring the structure to plasma adhesion receptors present on the membrane [92]. Hence, downregulation of filamin B β can possibly bring an impairment to cell structure and growth. Nevertheless, after fractionation, a big change in protein expression was seen for each fraction. This is especially true for SMHF as SMHF not only elevated CCT-β and -δ levels, but other CCT subunits like CCT- γ, ε, and η were also increased. SMHF also increased both the expression levels of cofilin 1, profilin 1, and phosphoserine aminotransferase. This could be an indication that SMHF might be able to counteract UVB damage by activating proteins needed in the wound healing process, despite not increasing myosin light polypeptide 6 and 9 expression levels. Further analysis on SMHF also showed that the fraction had additionally increased CAP1, filamin A, ezrin, keratin type I cytoskeletal 14, keratin II cytoskeletal 8, alpha-actinin 1, annexin A1, and LASP-1. These proteins that were elevated in expression have been ascribed as regulators of the actin cytoskeleton dynamics and some might potentially aid in the positive growth and proliferation of cells. In the case of filamin A, the lack of it was stated to cause a defect in the formation of cell junctions in filamin A null embryonic mice, while alpha-actinin 1 initiates cell motility through the regulation of focal adhesions, β4 integrin localization, and actin cytoskeleton organization [93,94]. Furthermore, the exposure of cell growth factors had shown stimulation of LASP 1 re-localization from the cell periphery to focal adhesion during cell migration, indicating its importance in cell migration [95]. On the other hand, even though annexin A1 is involved in many cellular processes, it was also reported to induce migration in fibroblast cells [96]. Finally, type I and II keratins are intermediate filaments and through post-translational modifications, they regulate cytoskeletal reorganization, while ezrin acts as linkers between the actin cytoskeleton and plasma membrane [97,98]. The other extract and fractions, besides SMEAF increasing the expression of keratin type I cytoskeletal 8, showed no upregulation in any of the proteins mentioned, suggesting that either they have very little or negative involvement in cell growth, proliferation, and migration after UVB irradiation. Although SMEAF increased the expression of keratin type I cytoskeletal 8, it also downregulated the expression of fascin, which is an actin bundling protein that has been reported to be involved in cell motility and invasion in cancer cell lines [99]. Therefore, due to the conflicting contradictory data, more studies are needed to truly understand the mechanism behind the molecular changes caused by SMEAF. SMWF, on the other hand, continues to downregulate many proteins involved in cell growth, proliferation, and migration, as can be seen in Figure 3, potentially suppressing the epidermis’s ability to heal and repair itself after UVB irradiation.

#### 2.2.6. SMHF Upregulates Proteins Involved in Cell Glycolysis

To maintain homeostasis and perform cellular maintenance such as DNA repair, protein turnover, transcription, and translation, etc., a considerable amount of energy is required [100]. Hence, changes in the glycolysis process are also important to take note as it could determine cell survival. In UVB control cells, phosphoglycerate kinase 1 was decreased, but this effect was attenuated by SMHF. In addition, SMHF also elevated the protein levels of malate dehydrogenase cytoplasmic, glucose-6-phosphate isomerase, triosephosphate isomerase, and ATP synthase subunit β. All these proteins listed are proteins that are involved in the glycolysis process with ATP synthase as the producer of ATP [101,102,103,104,105,106]. The upregulation of these proteins coincides with the data obtained for SMHF, whereby SMHF treated cells showed an increase in proteins involved in DNA repair and maintenance, RNA transcription and translation, protein processing after UVB irradiation. This further supports the possibility of SMHF attenuating the damage induced by UVB. Once again, no significant changes in these proteins were seen in SMCE and SMEAF. However, SMWF displayed downregulation in phosphoglycerate kinase 1, malate dehydrogenase cytoplasmic, glucose-6-phosphate isomerase, triosephosphate isomerase, and ATP synthase subunit β, suggesting that the glycolysis process may be suppressed by SMWF.

#### 2.2.7. Dysregulation of Gene Expression in HaCaT Cells after UVB Irradiation

**Figure 4:**
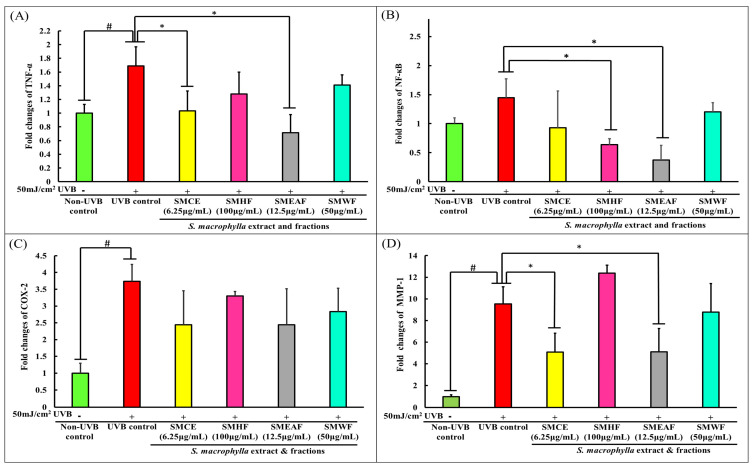

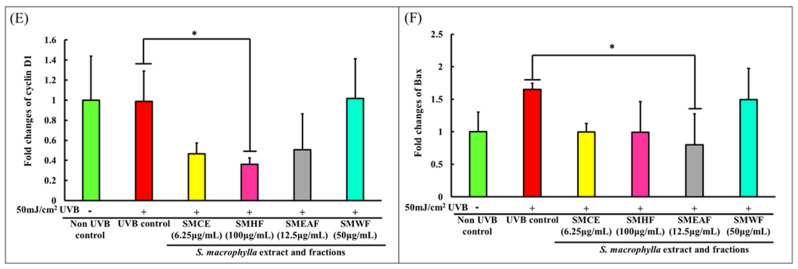
Gene expression changes in HaCaT cells after UVB irradiation and treatment with *S. macrophylla* extract and fractions. HaCaT cells were treated with SMCE, SMHF, SMEAF, and SMWF (at indicated concentrations) before being irradiated with 50 mJ/cm^2^ UVB immediately. After irradiation, the extract was removed and the cells were left to incubate for 24 h before mRNA extraction. The fold changes in (**A**) TNF-α, (**B**) NF-κB, (**C**) COX-2, (**D**) MMP-1 (**E**) cyclin D1, and (**F**) Bax mRNA expression were analyzed using qPCR. Data were expressed as mean ± standard deviation. (*n* ≥ 3; *p*-value ≤ 0.05; # symbolizes significant difference between non-UVB and UVB control; * symbolizes significant difference between UVB control and sample).

Besides delving deep into UVB-induced dynamic proteomic dysregulation, the modulatory effect of UVB on inflammatory and collagen degrading genes was also investigated. Here, we investigated three inflammatory markers: TNF-α, NF-κB, and COX-2, and a collagen degrading marker, MMP-1. Skin exposure to UVB induces the upregulation of ROS, which stimulates the cascade of inflammatory response such as the expression of NF-κB [107]. In turn, NF-κB then activates the production of proinflammatory cytokines, like TNF-α [108]. According to Yeo et al. [109], the increase of TNF-α can upregulate the binding of early growth response-1 (EGR-1) to the MMP-1 promoter through activation of extracellular signal-regulated kinase (ERK)1/2, JNK, and p38 kinase, ultimately increasing the expression of the MMP-1 protein. Furthermore, the overexpression of TNF-α can also activate the ASK1-mediated pathway, which prolongs the prolonged activation of JNK and p38 [110], thus, suggesting an equally prolonged expression of MMP-1 in the cell. On another note, NF-κB can also directly affect the production of MMP-1 as it has been discovered that the MMP-1 promoter also contains NF-κB binding sites [111,112]. A study done by Elliott, Coon, Hays, Stadheim, and Vincenti [111] had shown that NF-κB1 homodimers, together with IL-1β and Bcl-3, can activate the MMP-1 transcription. Finally, the expression of COX-2 is mediated by NF-κB and its expression has been linked with UVB-induced cell inflammation, death, and skin carcinogenesis [113,114]. As per data procured, UVB control cells displayed a significant increase in TNF-α, NF-κB, COX-2, and MMP-1 expression level by 1.69, 1.44, 3.74, and 9.54-fold, respectively, compared to non-UVB control cells (Figure 4A–D). Nevertheless, SMCE was able to negatively regulate the expression of TNF-α to a 1.03-fold change and MMP-1 to a 5.07-fold change. On the other hand, SMHF was only able to downregulate NF-κB (0.64-fold), while SMEAF significantly decreased TNF-α, NF-κB, and MMP-1 levels by 0.72, 0.37, and 5.11-fold change, accordingly. Aside from that, SMWF displayed no significant changes in the expression of all four genes compared to the UVB control. This inability of SMWF to suppress all four genes coincides with the proteomics data obtained, which is that SMWF is unable to attenuate UVB-induced cell death. Regardless, none of the extract and fraction were able to significantly decrease COX-2 transcription levels, although SMCE and SMEAF showed slightly lowered expressions.

The implications of SMCE, SMEAF, and SMHF’s ability to regulate these genes are important as these genes not only affect cell survival, but also the photoaging of the skin. Hence, the inhibition of SMEAF in the expression of NF-κB, TNF-α, and MMP-1 suggests that SMEAF can not only inhibit the UVB- induced inflammatory process, but is also able to suppress collagen degradation. SMCE also displayed the same aptitude as SMEAF, even though it was only able to slightly suppress the expression of NF-κB. However, in conjunction with the proteomics studies, even though SMHF significantly decreased the expression of NF-κB and significantly increased the expression of PRDX-1 (an inhibitor to the ASK-1 activity), it was not sufficient to suppress the expression of MMP-1. Based on this outcome, it might be suggested that the inability of SMHF to decrease MMP-1 expression could be due to its inability to significantly suppress the expression of TNF-α, showing the importance of TNF-α in the role of MMP-1 expression.

Other than the changes in inflammatory and collagen degradation, the effect of *S. macrophylla* against UVB-induced cell cycle arrest and apoptosis were also investigated. Subsequently, our study on cell cycle changes revealed that cyclin D1 experienced no changes in expression 24 h after 50 mJ/cm^2^ UVB irradiation (Figure 4E). However, treatment with SMHF significantly decreased the expression of cyclin D1 compared to the UVB control cells. Both SMCE and SMEAF also decreased the expression of cyclin D1, although not significantly, while cells treated with SMWF showed no changes in the expression of cyclin D1. In the process of cell cycle, the coordination of CDK, CDK inhibitor, and cyclin expressions are essential to ensure continuous cell proliferation, and among the cell cycle related genes, both CDK2/cyclinE1 and CDK4/cyclinD1 complexes have a role to play in the G1/S phase transition, while CDK1 and cyclin B1 is responsible for the transition of cells from the G2 to M phase [115]. However, under environmental stresses, the progression of cell cycle can be inhibited, thus halting cell proliferation [116]. According to Han and He [117], cell cycle progression of keratinocytes is UVB dose-dependent. All cells exposed to UVB were significantly arrested in the S phase 18 h after exposure. They also displayed an increase in cyclin D1 expression at the 3 h time point and decreased expression at the 6 h timepoint during the S phase. This increase in cyclin D1 was reported to be due to the activation of the AKT, ERK, and EGFR pathways, which in turn are activated by MMP. After 48 h, cells exposed to lower doses of UVB were seen to successfully exit the S phase and returned to the G0-G1 phase, but those that were exposed with higher doses of UVB stayed longer in the S phase. The arrest of cells by UVB in the S phase is most likely due to the increase in cyclin D1 expression as Yang et al. [118] reported that it is necessary for cyclin D1 levels to be low in the S phase to allow for efficient DNA synthesis. As UVB has been known to cause damage to DNA, it could be possible that the cells were arrested to inhibit DNA synthesis. In the case of SMHF treated cells, the decrease in cyclin D1 expression coincides with the data obtained from LC-MS/MS as SMHF was shown to upregulate proteins that are involved in DNA repair and maintenance. With the combination of increased DNA repair proteins and decreased cyclin D1 expression, it could be possible that SMHF treated cells are able to actively repair and synthesize DNA. However, this claim needs to be further confirmed with further tests. As for SMCE and SMEAF, the decrease in cyclin D1 could be due to their ability to suppress MMP-1 expression, but this has yet to be confirmed.

**Figure 5:**
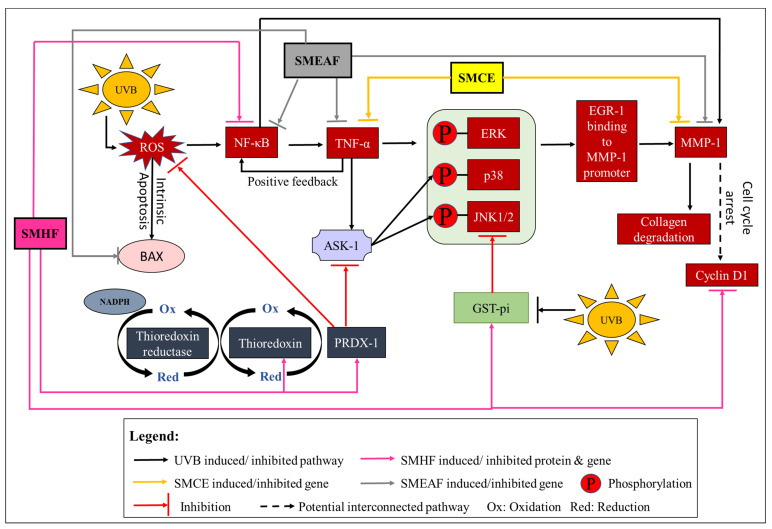
Activation of the redox system, cell inflammation, collagen degradation, cell cycle arrest and intrinsic apoptosis by UVB and the effect SMCE, SMHF, and SMEAF treatment has on the irradiated keratinocyte cells.

In the apoptotic pathway, we focused on the expression of Bcl-2-associated X protein (Bax) as it plays an important role in forming the apoptotic pores at the mitochondrial outer membrane. When the apoptotic pores form, it is then at this stage where it is considered as the point of no return for the cells [119]. The permeabilization of the mitochondria outer membrane will then lead to the release of cytochrome C and the activation of caspases involved in the intrinsic apoptotic pathway [119]. In our study, an increase in Bax expression was seen at 24 h in UVB control cells, as shown in Figure 4F. This increase in Bax in UVB control cells is expected as it was previously reported in several studies, indicating that the exposure of keratinocytes to UVB would ultimately lead to the activation of apoptosis [120,121]. However, when treated with SMCE, SMHF, and SMEAF, the expression of Bax was decreased. In addition, the decrease in Bax mRNA expression was significant for cells treated with SMEAF. Thus, this suggests that SMCE contains bioactive compounds that may be able to suppress UVB-induced apoptosis and after fractionation, SMHF and, especially, SMEAF, are the two fractions that contain the compounds responsible for suppressing Bax. In contrast, there were no changes in the expression of Bax when the irradiated cells were treated with SMWF. The lack of change in both cyclin D1 and Bax expression together with suppression of DNA repair proteins in SMWF treated cells might suggest that the cells may be experiencing cell cycle arrest and might be entering the apoptosis pathway. Even so, further analysis on the apoptotic pathway, extract fractionation, and continuous bio-guided assays is still necessary to identify the compound that is able to reverse or inhibit the effect of UVB. The changes in gene expression and its related pathway can be seen in Figure 5.

**Figure 6:**
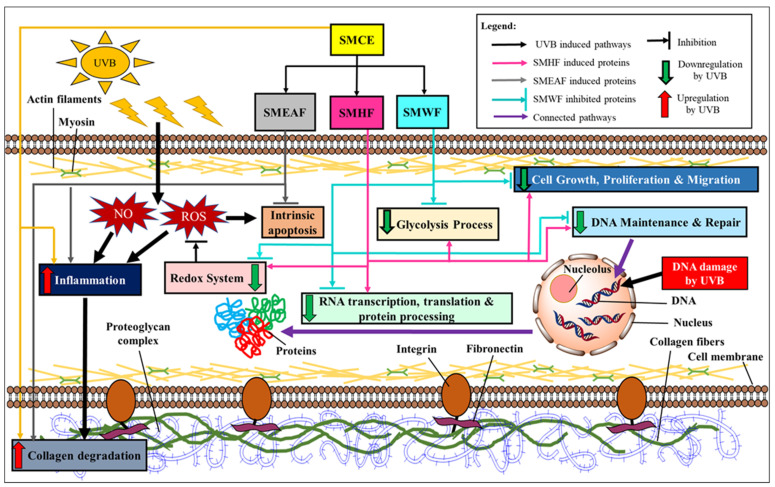
An overview of the effect of *S. macrophylla* extract and fractions against UVB-induced photodamage in keratinocyte cells.

Overall, these experiments showed that UVB does induce a massive change in both gene and protein expression, affecting their regulation and ultimately, cell survival. To provide an overview, Figure 6 depicts a summary of the gene and protein expression changes throughout the whole cell after UVB exposure. As the cells were irradiated with UVB, ROS and RNS were produced, while the redox system was inhibited. This leads to the production of inflammatory markers, which induces a cascade of reactions, leading to collagen degradation and subsequently, cell death. Furthermore, damage to the cell DNA also occurred, which in turn suppressed RNA transcription, translation, and protein processing. Subsequently, UVB exposure also decreased the expression of proteins involved in DNA maintenance and repair, inhibiting any form of repair on the UVB-induced CPD and pyrimidine-pyrimidone (6-4) photoproduct. Furthermore, the proteins involved in both the glycolysis process and cell growth, proliferation, and migration processes were also affected by UVB irradiation. All in all, the suppression of these genes and proteins will ultimately encourage the cell to begin cell death. However, this effect can be reversed with *S. macrophylla* as a treatment against UVB-induced photodamage. Although SMCE was only able to inhibit the production of inflammatory markers and subsequently collagen degradation itself, its fractions displayed a wider effect throughout the cells. SMHF exhibited a significant increase in a wide array of proteins that reverses the effect of UVB on many cell processes including the redox system, DNA synthesis and repair, glycolysis process, RNA to protein processes, and finally, it also induces cell growth, proliferation, and migration. On the other hand, SMEAF retained the same effect as SMCE but with the addition of being able to significantly inhibit Bax, indicating that the compounds responsible for the anti-inflammatory, anti-collagen degrading, and potentially anti-apoptotic activity may be within the SMEAF fraction. In comparison to the other fractions, SMWF was not only to be unable to inhibit the effect of UVB, but also additionally seems to further encourage cell death. Based on the data, it can be said that although SMCE can attenuate UVB-induced photodamage, its effect can be enhanced further via fractionation. This could be due to the removal of antagonistic compounds from the mixture, where in this case, SMHF showed an improved reversal effect against UVB damage while SMWF displayed the opposite effect. Hence, for future studies, further fractionation of the current existing fractions should be conducted to improve their efficacy against photoaging. Next, the creation of cosmetic formulation with *S. macrophylla* fractions as the active ingredient and in vivo human clinical testing of said formula on skin hydration, elasticity, sebum production, and pigmentation can also be conducted, as described by Tian et al. [122], Goh et al. [123], Adejokun and Dodou [124], and Mosquera et al. [125], to better study its anti-photoaging effect on the skin.

## 3. Materials and Methods

### 3.1. Plant Material and Extraction

Seeds of *S. macrophylla* (3 kg) were purchased from a local market and a voucher of the specimen (No. KLU46901) was deposited at the Herbarium of Institute of Biological Sciences, Faculty of Science, University of Malaya, Malaysia. To extract the bioactive compounds, the seeds were first dried and then ground finely before being soaked in ethanol at room temperature for 72 h. After filtration, the mixture was concentrated in a rotary vacuum evaporator at 40 °C to yield SMCE. Some portions of SMCE were then further processed to obtain SMHF using the hexane solvent. The liquid hexane fraction was separated from its insoluble residues via filtration, dried using anhydrous sodium sulfate, and concentrated again with the rotary vacuum evaporator at 40 °C. The insoluble residues were then subjected to a solvent–solvent portioning of ethyl acetate and water at a 1:1 ratio. After complete separation of both layers, SMEAF was evaporated using rotary vacuum evaporation while SMWF was freeze-dried with a freeze dryer [126].

### 3.2. Cell Line and Maintenance

The human keratinocyte (HaCaT) cell line (American Tissue Culture Center, Chapel Hill, NC, USA) was used to emulate the epidermal skin cells. They were maintained with culture medium of 1× high-glucose Dulbecco’s Modified Eagle Medium (DMEM), supplemented with GlutaMAX without HEPES (Gibco, Thermo Fisher, Waltham, MA, USA), 10.0% fetal bovine serum (Gibco, Thermo Fisher, Waltham, MA, USA), and 1.0% antibiotic/antimycotic solution (100 U/mL penicillin, 100 µg/mL streptomycin, and 25 µg/mL amphotericin B) (Gibco, Thermo Fisher, Waltham, MA, USA). The cells were incubated at 37 °C in 5% CO_2_ atmospheric conditions [127].

### 3.3. Cytotoxicity Assay

The cytotoxicity assay was conducted to determine the non-cytotoxic concentration of each extract and fraction on HaCaT cells. The cells were incubated with 0, 1.56, 3.125, 6.25, 12.5, 25, 25, 50, and 100 µg/mL of each extract and fraction for 24 h before its cell viability was assessed using the 3-(4,5-dimethylthiazol-2-Y1)-2,5-diphenyltetrazolium bromide (MTT) assay. In each well, 20 μL of MTT was added and the plate was then incubated for 2 h. After that, the solution was removed and100 µL of dimethyl sulfoxide (DMSO) was then added into each well. The absorbance was measured at 570 nm via a microplate reader and the percentage of cell viability was calculated after normalizing against the control cells.

### 3.4. UV Irradiation

To study the effect of UVB on keratinocyte cells, the cells were seeded in six well plates at a concentration of 300,000 cells/well. The following day, the cells were rinsed with phosphate buffered saline (PBS) and then treated with either 6.25 μg/mL SMCE, 100 μg/mL SMHF, 12.5 μg/mL SMEAF, or 50 μg/mL SMWF in 1.5 mL PBS per well, with 0.5% DMSO as the vehicle, before being irradiated with 50 mJ/cm^2^ of UVB using a Philip UVB Broadband TL 20W/12 phototherapy lamp (Philip, Amsterdam, The Netherlands) according to Mahendra et al. [128] with slight modifications. The UVB dose was measured using a Lutron UV light meter UV-340A (Lutron, Taipei, Taiwan). After irradiation, the PBS solution was removed and replaced with 3 mL of media in each well. Non-UVB and UVB control plates (untreated cells that were either irradiated or non-irradiated) were treated the same way as the sample plates. This included rinsing and treating the cells with 0.5% DMSO in 1.5 mL PBS per well for a similar amount of time before the PBS was replaced with media. The cells were incubated for 24 h before RNA or protein extraction.

### 3.5. Protein Expression Studies

#### 3.5.1. Preparation of Lysis Buffer

Fresh lysis buffer was prepared a day before protein extraction. The lysis buffer was prepared by combining 10 mM Tris solution with 0.1% Triton X. The pH of the solution was then adjusted to pH 7.4 using hydrochloric acid before being sterile filtered with a 0.22 µM cellulose acetate syringe filter (Sartorius, Göttingen, Germany) and stored at 4 °C.

#### 3.5.2. Protein Extraction

Cells were harvested using Tryple E (Gibco, Thermo Fisher, Waltham, MA, USA) and pelleted via centrifugation at 1000 rpm for 5 min. To completely remove the presence of media, the cells were rinsed and pelleted twice. Next, the PBS was removed and the pellet was resuspended in 80 µL of ice-cold lysis buffer. The mixture was then freeze–thaw for three cycles under these conditions: −152 °C, 10 min; 37 °C for 2–3 min. Subsequently, the mixture was centrifuged at 10,000× *g* at 4 °C for 15 min and the supernatant was collected and stored at −80 °C in low protein binding microcentrifuge tubes (Eppendorf, Hamburg, Germany).

#### 3.5.3. Bicinchoninic Acid (BCA) Protein Assay

Protein concentration was measured via a BCA Protein Assay Kit (Thermo Fisher, Waltham, MA, USA), according to the manufacturer’s instructions. In short, reagent A and B were mixed at a 50:1 ratio to create the working reagent. After that, aa 10 µL protein sample was added to the well with 200 µL working reagent. The plate was then incubated at 37 °C for 30 min before allowing to cool and measured at 562 nm. Later, the absorbance obtained was subtracted with the blank absorbance prior to calculating the concentration based on the standard curve. The standard curve was built on the increasing concentrations (0–1500 µg/mL) of bovine serum albumin (BSA) (Sigma, St. Louis, MI, USA).

#### 3.5.4. In-Solution Tryptic Digestion

Protein samples (10 mg/mL) were denatured in a solution containing 1 µL of 200 mM 1,4-dithiothreitol (DTT), 25 µL of 100 mM ammonium bicarbonate, and 25 µL trifluoroethanol (TFE). The mixture was mixed via vortexing and heated for 1 h at 60 °C. After incubation, 4 µL of 200 mM iodoacetamide (IAM) stock was added to the mixture to alkylate the proteins for 1 h in the dark at room temperature. Next, 1 µL of 200 mM DTT was added to quench the excess IAM and the mixture was again incubated for 1 h in the dark at room temperature. Subsequently, 300 µL of ultrapure water and 100 µL of 100 mM ammonium bicarbonate was added to the samples before following with 1 µL of 20 µg/mL MS grade trypsin (Thermoscientific, Waltham, MA, USA), which was reconstituted in 50 mM acetic acid. The mixture was incubated for 16 h at 37 °C for complete protein digestion. After that, 1 µL of formic acid was added to the mixture to stop the trypsin reaction. Finally, the samples were dried overnight in the MiniVac speed vacuum concentrator (Lobogene, Lillerød, Denmark) and stored at −20 °C.

#### 3.5.5. Protein Sample Desalting and Cleanup

The samples were cleaned and desalted before LCMS/MS analysis using a Pierce^®^ C18 Spin column (Thermoscientific, Waltham, MA, USA), according to the manufacturer’s instructions. After clean up and desalting, the sample was once again dried overnight in the MiniVac speed vacuum concentrator (Lobogene, Lillerød, Denmark) and stored at −20 °C.

#### 3.5.6. Analysis of Protein Samples with Nanoflow-Ultra High-Performance Chromatography-Tandem Mass Spectrometry (LC-MS/MS)

This method was done following the method described by Paudel et al. [129]. First, protein samples were dissolved in 30 µL 0.1% formic acid before centrifuging at 14,000 rpm for 10 min. Next, 1 µL of the sample was loaded into an Agilent C18, 300 Å large capacity chip (Agilent Technologies, Santa Clara, CA, USA), and the chip was mounted onto the Agilent 1200HPLC-Chip/MS interface, which was coupled with an Agilent 6500 iFunnel quadrupole-time of flight (Q-TOF) LC/MS system. The flow rate was set at 4 µL/min from an Agilent 1200 Series Capillary pump and 0.5 µL/min from an Agilent 1200 Series Nano Pump with solution A (0.1% formic acid in water) and solution B (90% acetonitrile and 0.1% formic acid in water). The samples were then eluted with multi-step gradients of 5–75% of solution B (30 min of 5–75% solution B, 9 min of 75% solution, and 8 min of 5–75% solution B). The ion polarity of Q-TOF was set at positive with a capillary voltage of 2050 V, the gas temperature at 325 °C, fragmentor voltage at 360 V, and finally, drying gas flow rate of 5 L/min. The spectra acquired were in auto MS/MS mode with an MS scan range of 110–3000 *m*/*z* and an MS/MS scan range of 50–3000 *m*/*z*. The precursor charge state selection and preference were fixed as doubly, triply, or more than triply charged state, with the exclusion of precursor 299/294457 *m*/*z* (Z = 1) and 1221.990637 *m*/*z* (Z = 1) (reference ions).

#### 3.5.7. Protein Identification and Differential Expression Studies with PEAKS Bioinformatics Software

To determine the protein identification and differential expression, label-free quantification (LFQ) was conducted using PEAKS studio 7.5 (Bioinformatics Solution Inc., Waterloo, ON, Canada) using the method as described by Paudel et al. (2020) with slight modification. Homo sapiens (Uniprot database) was used for homology search and protein identification. The carbamidomethylation was preset as a fixed modification with maximum missed cleavages at 3. Parent mass and fragment mass error tolerance were set at 0.1 Da with monoisotopic as the precursor mass search type. Trypsin was then selected as the digestion enzyme. The data filtering parameters were set at 1% false discover rate (FDR) with unique peptides ≥1. The LFQ parameters used were: retention time shift tolerance of 6 min, mass error tolerance of 20 ppm, and FDR threshold of 1%. The protein expression of the UVB control cells was compared against the non-UVB control cells, while samples treated with SMCE, SMHF, SMEAF, SMWF were compared against the UVB control cells using hierarchical clustering. The heat map was generated by setting the protein significance ≥13, which was the *p*-value of 0.05, fold change ≥1, and had at least one unique peptide. To calculate the significance, PEAKS Q was used and experimental bias was taken into account via automatic normalization of protein ratios in accordance with the total ion chromatogram (TIC) [129].

### 3.6. Quantitative Polymerase Chain Reaction (PCR) Analysis of Gene Expression Changes in UVB Irradiated Cells

Total mRNA was then collected using 300 μL of Trizol. The Trizol was then processed with a 1:5 ratio of chloroform to Trizol. The solution was vortexed and incubated for 3 min at room temperature before centrifuging at 13,500 rpm at 4 °C for 15 min. The upper colorless aqueous phase was extracted and mixed with 100% isopropyl alcohol at a 1:2 ratio. The mixture was mixed gently and incubated for 10 min at room temperature before centrifuging again at 13,500 rpm at 4 °C for 15 min. The supernatant was discarded carefully and the pellet was rinsed with 75% ethanol before centrifuging at 10,500 rpm for 5 min at 4 °C. The supernatant was discarded and the rinsing process was repeated. Finally, the pellet was air-dried for 5 min and dissolved in 10 μL of DEPC ultrapure water. The RNA concentration was measured at A260/A280. Total mRNA that was collected was converted to cDNA using a High Capacity cDNA Reverse Transcription Kit (Applied Biosystem, San Francisco, CA, USA) and cDNA was prepared for gene expression reading using the Power SYBR Green PCR Master Mix. qPCR analysis was done using the Step One Plus Real-Time PCR System (Applied Biosystem, San Francisco, CA, USA). Primers for 18S ribosomal RNA, TNF-α, NF-κB, COX-2, Bax, cyclin D1, and MMP-1 were either obtained from journals or designed using NCBI Primer Blast. The sequences of each primer can be seen in Table 2.

### 3.7. Statistical Analysis

All quantitative data were analyzed using the SPSS statistical analysis software and the results were expressed as mean ± standard deviation (SD). One-way Analysis of Variance (ANOVA) and Tukey post-hoc were used to determine significant data. The significant value was set at *p* ≤ 0.05.

## 4. Conclusions

In summary, a wide view of the adverse effect UVB has on a cellular level was portrayed through this research. The range of impact of UVB from the activation of the redox system and skin inflammation, to the suppression of protein synthesis, inhibition of cell growth and repair, induction of DNA damage, signaling of collagen degradation, and finally cell death are processes that cosmetic companies constantly battle with to keep their clients looking young. When tested with the *S. macrophylla* extract and fractions, two fractions, namely SMEAF and SMHF, exhibited potential photoprotective properties. Functioning via two completely different mechanisms, SMEAF showcased its ability to suppress inflammation, collagen degradation, and potentially the intrinsic apoptosis pathway on a cellular level while SMHF displayed its photoprotective properties through its involvement in the redox system, DNA repair, RNA transcription, protein maintenance and synthesis, cell growth, migration and proliferation, and cell glycolysis processes. Thus, as per the results, further analysis and fractionation of SMHF and SMEAF are warranted toward the making of anti-photoaging cosmetic active ingredients. Furthermore, in vivo human clinical studies with *S. macrophylla* cosmetic formulations can be conducted in the future to better evaluate the effect of *S. macrophylla* against photoaging.

## Figures and Tables

**Table 1:** A proteomic study of molecular changes within HaCaT cells after UVB irradiation and treatment with *S. macrophylla* extract and fractions.

No.	Name of Protein (Uniprot ID)	Comparison Ratio of Untreated Controls	Comparison Ratio of UVB Control vs. *S. macrophylla* Treatment + UVB Samples			
		Non-UVB: UVB	UVB: SMCE + UVB	UVB: SMHF + UVB	UVB: SMEAF + UVB	UVB: SMWF + UVB
1	Chaperonin containing TCP1 subunit 2 β isoform CRA_b (CCT-β) (V9HW96)	1.00:0.49 (↓)	N/S ^1^	1.00:1.41 (↑)	N/S	N/S
2	T-complex protein 1 subunit delta (CCT-δ) (A8K3C3)	1.00:0.47 (↓)	N/S	1.00:1.71 (↑)	N/S	1.00:0.52 (↓)
3	T-complex protein 1 subunit gamma (CCT-γ) (B3KX11)	N/S	N/S	1.00:1.58 (↑)	N/S	N/S
4	T-complex protein 1 subunit eta (CCT-η) (Q99832)	N/S	N/S	1.00:1.92 (↑)	N/S	N/S
5	T-complex protein 1 subunit epsilon (CCT-ε) (P48643)	N/S	N/S	1.00:2.39 (↑)	N/S	N/S
6	Ribosomal protein L12 variant (Q59FI9)	1.00:0.34 (↓)	N/S	N/S	N/S	N/S
7	Ribosomal protein S8 (Q9BS10)	N/S	N/S	1.00:1.90 (↑)	N/S	N/S
8	40S ribosomal protein SA (C9J9K3)	N/S	N/S	1.00:1.87 (↑)	N/S	N/S
9	40S ribosomal protein S5 (M0QZN2)	1.00:0.32 (↓)	N/S	N/S	N/S	1.00:0.32 (↓)
10	40S ribosomal protein S7 (P62081)	1.00:0.45 (↓)	N/S	N/S	N/S	1.00:0.29 (↓)
11	40S ribosomal protein S10 (P46783)	1.00:0.33 (↓)	N/S	N/S	N/S	1.00:0.31 (↓)
12	40S ribosomal protein S16 (M0R210 or M0R3H0)	1.00:0.36 (↓)	N/S	N/S	N/S	1.00:0.51 (↓)
13	40S ribosomal protein S25 (P62851)	1.00:0.48 (↓)	N/S	N/S	N/S	1.00:0.50 (↓)
14	60S ribosomal protein L6 (Q8N5Z7)	1.00:0.31 (↓)	N/S	N/S	N/S	N/S
15	60S ribosomal protein L18 (F8VUA6)	1.00:0.51 (↓)	1.00:0.22 (↓)	1.00:0.68 (↓)	N/S	1.00:0.44 (↓)
16	60S ribosomal protein L22 (K7EJT5)	1.00:0.37 (↓)	N/S	1.00:1.71 (↑)	N/S	N/S
17	60S ribosomal protein L24 (C9JXB8)	1.00:0.48 (↓)	N/S	N/S	N/S	N/S
18	60S ribosomal protein L29 (A0A024R326)	1.00:0.50 (↓)	N/S	N/S	N/S	1.00:0.39 (↓)
19	60s acidic ribosomal protein P0 (Q53HW2)	N/S	N/S	N/S	N/S	1.00:0.40 (↓)
20	60s acidic ribosomal protein P2 (P05387)	N/S	N/S	1.00:1.39 (↑)	N/S	1.00:0.55 (↓)
21	Albumin (F6KPG5)	1.00:2.66 (↑)	N/S	N/S	N/S	1.00:0.41 (↓)
22	chloride intracellular channel (A0A1U9 × 8Y4)	N/S	N/S	N/S	N/S	1.00:0.63 (↓)
23	chloride intracellular channel 1 (CLIC1) (O00299)	1.00:0.56 (↓)	N/S	1.00:1.55 (↑)	N/S	N/S
24	Cofilin 1 (V9HWI5)	1.00:0.61 (↓)	N/S	1.00:1.38 (↑)	N/S	1.00:0.63 (↓)
25	Elongation factor 1-alpha (Q6IPN6 or Q53HM9)	1.00:0.36 (↓)	N/S	1.00:1.88 (↑)	N/S	N/S
26	Elongation factor 1-delta (A0A087X1X7 or E9PN91)	1.00:0.33 (↓)	N/S	1.00:1.51 (↑)	1.00:1.49 (↑)	1.00:0.66 (↓)
27	Eukaryotic translation elongation factor 2 (eEF2) (Epididymis secretory sperm binding protein) (A0A384N6H1)	1.00:0.55 (↓)	N/S	1.00:1.67 (↑)	N/S	1.00:0.41 (↓)
28	Glycine-tRNA ligase (P41250)	1.00:0.28 (↓)	N/S	1.00:7.22 (↑)	N/S	N/S
29	Heat shock protein (HSP)-10 kDa (Chaperonin 10) (Epididymis secretory sperm binding protein) (A0A384N6A4)	N/S	N/S	1.00:1.48 (↑)	N/S	1.00:0.53 (↓)
30	HSP-60 kDa mitochondrial (P10809)	N/S	N/S	N/S	N/S	1.00:0.52 (↓)
31	HSP-70 kDa protein 1A variant (Q59EJ3)	N/S	N/S	N/S	N/S	1.00:0.54 (↓)
32	HSP-70 kDa protein 4 (Q59GF8)	N/S	N/S	1.00:1.36 (↑)	N/S	N/S
33	HSP-70 family protein 5 (Epididymis secretory sperm binding protein Li 89n) (78 kDa glucose-regulated protein) (V9HWB4)	N/S	N/S	N/S	N/S	1.00:0.54 (↓)
34	HSP-70 kDa protein 8, isoform CRA_a (Epididymis luminal protein 33) (V9HW22)	1.00:0.52 (↓)	N/S	1.00:1.50 (↑)	N/S	N/S
35	HSP-70 kDa protein 9 (75 kDa glucose-regulated protein) (B7Z4V2)	N/S	N/S	1.00:1.35 (↑)	N/S	N/S
36	HSP 90α (P07900)	1.00:0.60 (↓)	N/S	1.00:1.51 (↑)	N/S	1.00:0.51 (↓)
37	Gluthathione S-transferase (GST)-pi (Epididymis secretory protein Li 22) (V9HWE9)	1.00:0.63 (↓)	N/S	1.00:1.45 (↑)	N/S	1.00:0.58 (↓)
38	Poly (RC) binding protein 1 (Epididymis secretory protein Li 85) (Q53SS8)	1.00:0.32 (↓)	N/S	1.00:2.10 (↑)	N/S	1.00:0.56 (↓)
39	Histone H2A type 1-A (Q96QV6)	N/S	N/S	N/S	1.00:1.41 (↑)	N/S
40	Histone H2A type 2-B (Q8IUE6)	1.00:2.75 (↑)	N/S	1.00:1.66 (↑)	N/S	1.00:0.56 (↓)
41	Histone H2B (A8K9J7 or B4DR52)	1.00:2.86 (↑)	N/S	N/S	N/S	1.00:0.40 (↓)
42	Histone H1.2 (P16403)	N/S	N/S	1.00:1.69 (↑)	1.00:1.41 (↑)	1.00:0.62 (↓)
43	Histone H1.5 (P16401)	N/S	N/S	1.00:2.01 (↑)	N/S	N/S
44	Histone H4 (B2R4R0)	N/S	N/S	N/S	N/S	1.00:0.50 (↓)
45	Myosin light polypeptide 6 (F8W1R7)	1.00:0.43 (↓)	N/S	N/S	N/S	N/S
46	Myosin 9 (P35579)	1.00:0.48 (↓)	N/S	N/S	N/S	1.00:0.52 (↓)
47	Nucleophosmin (Nucleolar phosphoprotein B23 numatrin) isoform CRA_f (A0A0S2Z4G7)	1.00:0.71 (↓)	N/S	1.00:1.34 (↑)	N/S	1.00:0.43 (↓)
48	Peptidyl-prolyl cis-trans isomerase (V9HWF5)	1.00:0.56 (↓)	N/S	N/S	N/S	1.00:0.43 (↓)
49	PRDX-1 (A0A384NPQ2 or A0A0A0MSI0)	1.00:0.57 (↓)	N/S	1.00:1.67 (↑)	N/S	1.00:0.43 (↓)
50	PRDX-2 (B4DF70)	N/S	N/S	N/S	N/S	1.00:0.51 (↓)
51	PRDX-3 (Thioredoxin-dependent peroxide reductase mitochondrial) (P30048)	N/S	N/S	1.00:0.58 (↓)	1.00:0.73 (↓)	N/S
52	PRDX-6 (P30041)	1.00:0.58 (↓)	N/S	N/S	N/S	1.00:0.42 (↓)
53	Phosphoglycerate kinase 1 (P00558)	1.00:0.61 (↓)	N/S	1.00:1.58 (↑)	N/S	1.00:0.50 (↓)
54	Phosphoserine aminotransferase (Q9Y617)	1.00:0.56 (↓)	N/S	1.00:1.46 (↑)	N/S	1.00:0.39 (↓)
55	Profilin-1 (P07737)	1.00:0.63 (↓)	N/S	1.00:1.42 (↑)	N/S	1.00:0.56 (↓)
56	PDI (A0A024R8S5)	N/S	N/S	1.00:1.52 (↑)	N/S	1.00:0.63 (↓)
57	PDI-A3 (P30101)	N/S	N/S	1.00:1.66 (↑)	1.00:0.76 (↓)	N/S
58	PDI-A4 (P13667)	N/S	N/S	1.00:2.30 (↑)	N/S	1.00:0.60 (↓)
59	PDI-A6 (Endoplasmic reticulum protein 5) (Q15084)	1.00:1.74 (↑)	N/S	N/S	N/S	1.00:0.43 (↓)
60	Protein S100 (A0A590UJ49 or B2R5H0)	1.00:0.30 (↓)	N/S	1.00:1.47 (↑)	N/S	1.00:0.33 (↓)
61	Protein-arginine deiminase type-1 (Q9ULC6)	1.00:15.79 (↑)	N/S	N/S	N/S	1.00:0.21 (↓)
62	Pyruvate kinase PKM (P14618)	1.00:0.67 (↓)	N/S	1.00:1.33 (↑)	N/S	1.00:0.48 (↓)
63	RACK-1 (P63244 or D6RF23)	1.00:0.22 (↓)	N/S	N/S	N/S	1.00:9.32 (↑)
64	Signal recognition particle 14 kDa protein (SRP-14) (P37108)	1.00:0.33 (↓)	N/S	N/S	N/S	N/S
65	SYNCRIP protein (Q05CK9)	1.00:0.48 (↓)	N/S	N/S	N/S	N/S
66	Transketolase (B3KSI4)	1.00:0.52 (↓)	N/S	N/S	N/S	N/S
67	D-3-phosphoglycerate dehydrogenase (B3KSC3)	1.00:0.50 (↓)	N/S	N/S	N/S	N/S
68	Fumarate hydratase (Epididymis secretory sperm binding protein) (A0A0S2Z4C3)	N/S	1.00:2.02 (↑)	1.00:2.43 (↑)	N/S	N/S
69	Annexin A1 (Q5TZZ9)	N/S	N/S	1.00:1.46 (↑)	N/S	1.00:0.43 (↓)
70	Annexin A2 (A0A024R5Z7)	N/S	N/S	N/S	N/S	1.00:0.46 (↓)
71	Annexin A3 (P12429)	N/S	1.00:0.63 (↓)	N/S	1.00:0.52 (↓)	1.00:0.42 (↓)
72	Annexin A5 (P08758)	N/S	N/S	N/S	N/S	1.00:0.42 (↓)
73	Filamin A (Q60FE6)	N/S	N/S	1.00:1.54 (↑)	N/S	1.00:0.36 (↓)
74	Filamin B β (Actin binding protein 278) isoform CRA_a (A0A024R321)	N/S	1.00:0.44 (↓)	N/S	N/S	N/S
75	3-phosphoglycerate dehydrogenase (Q9UMY2 or Q9UMY3)	N/S	N/S	1.00:2.27 (↑)	N/S	1.00:0.26 (↓)
76	14-3-3 protein α/β (P31946)	N/S	N/S	N/S	N/S	1.00:0.60 (↓)
77	14-3-3 protein σ (P31947)	N/S	N/S	1.00:1.97 (↑)	N/S	1.00:0.47 (↓)
78	14-3-3 protein γ (P61981)	N/S	N/S	N/S	N/S	1.00:0.18 (↓)
79	14-3-3 protein ε (P62258)	N/S	N/S	N/S	N/S	1.00:0.38 (↓)
80	Ubiquitin-activating enzyme E1 (Testicular secretory protein Li 63) (A0A024R1A3)	N/S	N/S	1.00:1.84 (↑)	N/S	N/S
81	AHNAK (Desmoyokin) (Q09666)	N/S	N/S	1.00:0.55 (↓)	N/S	N/S
82	Keratin type I cytoskeletal 14 (P02533)	N/S	N/S	1.00:1.86 (↑)	1.00:1.88 (↑)	N/S
83	Keratin type II cytoskeletal 8 (P05787)	N/S	N/S	1.00:1.48 (↑)	N/S	1.00:0.52 (↓)
84	Heterogeneous nuclear ribonucleoprotein (hnRP) D0 (D6RF44)	N/S	N/S	1.00:1.74 (↑)	N/S	N/S
85	hnRP K (B4DFF1 or B4DUQ1)	N/S	N/S	1.00:1.54 (↑)	N/S	1.00:0.51 (↓)
86	Adenylyl cyclase-associated protein (CAP1) (B4DI38)	N/S	N/S	1.00:1.54 (↑)	N/S	1.00:0.38 (↓)
87	Reticulon-4 (Q6IPN0 or Q9NQC3)	N/S	N/S	1.00:1.70 (↑)	N/S	1.00:0.64 (↓)
88	Malate dehydrogenase cytoplasmic (P40925)	N/S	N/S	1.00:1.70 (↑)	N/S	1.00:0.72 (↓)
89	Transketolase (A0A0B4J1R6)	N/S	N/S	1.00:1.62 (↑)	N/S	1.00:0.34 (↓)
90	Glucose-6-phosphate isomerase (P06744)	N/S	N/S	1.00:1.61 (↑)	N/S	1.00:0.36 (↓)
91	Proteasome subunit alpha type (Q53GF5 or H0YLC2)	N/S	N/S	1.00:1.45 (↑)	N/S	1.00:0.32 (↓)
92	Proteasome activator complex subunit 2 (Q86SZ7)	N/S	N/S	1.00:1.63 (↑)	N/S	N/S
93	Proteasome subunit β type-2 (P49721)	N/S	N/S	N/S	N/S	1.00:0.42 (↓)
94	Proteasome subunit β type-3 (A0A087WXQ8)	N/S	N/S	1.00:1.59 (↑)	N/S	N/S
95	Glyceraldehyde-3-phosphate dehydrogenase (GAPDH) (P04406)	N/S	N/S	1.00:1.57 (↑)	N/S	1.00:0.47 (↓)
96	Proliferating cell nuclear antigen (PCNA) (P12004 or Q6FHF5)	N/S	N/S	1.00:1.55 (↑)	N/S	1.00:0.53 (↓)
97	Exportin-2 (P55060)	N/S	N/S	1.00:1.62 (↑)	1.00:1.33 (↑)	N/S
98	Serpin peptidase inhibitor clade B (Ovalbumin) member 5 isoform CRA_b (SERPINB5) (A0A024R2B6)	N/S	N/S	1.00:1.52 (↑)	N/S	1.00:0.51 (↓)
99	Collagen-binding protein (Serpin H1) (B4DN87)	N/S	N/S	1.00:1.80 (↑)	N/S	N/S
100	Ezrin (Q6NUR7)	N/S	N/S	1.00:1.51 (↑)	N/S	1.00:0.19 (↓)
101	Tubulin beta chain (A0A384NYT8)	N/S	N/S	1.00:1.47 (↑)	N/S	1.00:0.49 (↓)
102	Thioredoxin (P10599)	N/S	N/S	1.00:1.40 (↑)	N/S	1.00:0.54 (↓)
103	Thioredoxin domain-containing protein 17 (TXNDC17) (Testicular tissue protein Li 214) (A0A140VJY7)	N/S	N/S	1.00:1.47 (↑)	N/S	N/S
104	Alpha-enolase (P06733)	N/S	N/S	1.00:1.46 (↑)	N/S	1.00:0.46 (↓)
105	Triosephosphate isomerase (V9HWK1)	N/S	N/S	1.00:1.45 (↑)	N/S	1.00:0.44 (↓)
106	Alpha-actinin-1 (P12814)	N/S	N/S	1.00:1.44 (↑)	N/S	N/S
107	Calreticulin variant (Q53G71)	N/S	N/S	1.00:1.44 (↑)	N/S	1.00:0.42 (↓)
108	L-lactate dehydrogenase A (V9HWB9)	N/S	N/S	1.00:1.38 (↑)	N/S	1.00:0.35 (↓)
109	L-lactate dehydrogenase B (Q5U077)	N/S	N/S	1.00:1.39 (↑)	N/S	1.00:0.42 (↓)
110	ATP synthase subunit α, mitochondrial (V9HW26)	N/S	N/S	N/S	N/S	1.00:0.44 (↓)
111	ATP synthase subunit β, mitochondrial (P06576)	N/S	N/S	1.00:1.37 (↑)	N/S	1.00:0.59 (↓)
112	Endoplasmin (P14625)	N/S	N/S	1.00:1.36 (↑)	N/S	1.00:0.57 (↓)
113	Nucleosome assembly protein 1-like 1 (F8W020)	N/S	N/S	1.00:1.35 (↑)	N/S	N/S
114	Neutral amino acid transporter B(0) (Q15758)	N/S	N/S	1.00:1.38 (↑)	N/S	1.00:0.48 (↓)
115	LIM and SH3 domain protein 1 (LASP-1) (A8K1D2)	N/S	N/S	1.00:1.36 (↑)	N/S	N/S
116	Polyubiquitin-C (F5GYU3)	N/S	N/S	N/S	1.00:0.56 (↓)	1.00:0.33 (↓)
117	HNRPCL1 protein (Q6PKD2)	N/S	N/S	N/S	1.00:0.46 (↓)	N/S
118	Fascin (B3KTA3)	N/S	N/S	N/S	1.00:0.64 (↓)	1.00:0.36 (↓)
119	Cathepsin D (A0A1B0GW44)	N/S	N/S	N/S	1.00:0.70 (↓)	1.00:0.49 (↓)
120	Prothymosin alpha (Q15203)	N/S	N/S	N/S	1.00:0.20 (↓)	1.00:0.27 (↓)
121	GTP-binding nuclear protein Ran (P62826)	N/S	N/S	N/S	1.00:0.72 (↓)	1.00:0.41 (↓)
122	NOLC1 (B2RAU8)	N/S	N/S	N/S	1.00:1.39 (↑)	N/S
123	Protein kinase C substrate 80K-H isoform 1 (A0A0S2Z4D8)	N/S	N/S	N/S	1.00:1.35 (↑)	N/S
124	Actin cytoplasmic 1 (P60709)	N/S	N/S	N/S	N/S	1.00:0.25 (↓)
125	Actin cytoplasmic 2 (P63261)	N/S	N/S	N/S	N/S	1.00:0.47 (↓)
126	Rab GDP dissociation inhibitor β (P50395)	N/S	N/S	N/S	N/S	1.00:0.34 (↓)
127	Eukaryotic translation initiation factor 5A (I3L397)	N/S	N/S	N/S	N/S	1.00:0.39 (↓)
128	Phosphoglycerate mutase (Q6FHU2)	N/S	N/S	N/S	N/S	1.00:0.43 (↓)
129	Neutral alpha-glucosidase AB (Epididymis secretory sperm binding protein Li 164nA) (V9HWJ0)	N/S	N/S	N/S	N/S	1.00:0.33 (↓)
130	Adenosylhomocysteinase (P23526)	N/S	N/S	N/S	N/S	1.00:0.44 (↓)
131	4F2 cell-surface antigen heavy chain (F5GZS6)	N/S	N/S	N/S	N/S	1.00:0.44 (↓)
132	Protein SET (A0A0C4DFV9)	N/S	N/S	N/S	N/S	1.00:0.45 (↓)
133	Inorganic pyrophosphatase (Epididymis secretory sperm binding protein Li 66p) (V9HWB5)	N/S	N/S	N/S	N/S	1.00:0.42 (↓)
134	Aspartate aminotransferase (B3KUZ8)	N/S	N/S	N/S	N/S	1.00:0.47 (↓)
135	Plasminogen activator inhibitor 2 (P05120)	N/S	N/S	N/S	N/S	1.00:0.52 (↓)
136	Acidic leucine-rich nuclear phosphoprotein 32 family member A (ANP32B) (H0YN26)	N/S	N/S	N/S	N/S	1.00:0.52 (↓)
137	Complement component 1 Q subcomponent-binding protein mitochondrial (Q07021)	N/S	N/S	N/S	N/S	1.00:0.52 (↓)
138	Fructose-bisphosphate aldolase A (P04075)	N/S	N/S	N/S	N/S	1.00:0.53 (↓)
139	Transaldolase (A0A140VK56)	N/S	N/S	N/S	N/S	1.00:0.53 (↓)
140	Macrophage migration inhibitory factor (P14174)	N/S	N/S	N/S	N/S	1.00:0.55 (↓)
141	Endoplasmic reticulum resident protein 29 (P30040)	N/S	N/S	N/S	N/S	1.00:0.49 (↓)
142	Calmodulin-2 (P0DP24)	N/S	N/S	N/S	N/S	1.00:0.59 (↓)
143	RAN binding protein 1 isoform CRA_g (A0A140VK94)	N/S	N/S	N/S	N/S	1.00:0.59 (↓)
144	Vinculin isoform CRA_c (A0A024QZN4)	N/S	N/S	N/S	N/S	1.00:0.32 (↓)
145	Small ubiquitin-related modifier (A0A024R8S3)	N/S	N/S	N/S	N/S	1.00:0.37 (↓)
146	Calnexin (P27824)	N/S	N/S	N/S	N/S	1.00:0.61 (↓)
147	Nucleolin (P19338)	N/S	N/S	N/S	N/S	1.00:0.49 (↓)
148	RPLP1 protein (Q6ICQ4)	N/S	N/S	N/S	N/S	1.00:0.64 (↓)
149	FK506 binding protein 12 (Q1JUQ3)	N/S	N/S	N/S	N/S	1.00:0.12 (↓)
150	Lysosome-associated membrane glycoprotein 1 (B3KRY3)	N/S	N/S	N/S	N/S	1.00:0.26 (↓)
151	Transgelin-2 (P37802)	N/S	N/S	N/S	N/S	1.00:0.52 (↓)

^1^ N/S represents data that was not significant, while the upregulation and downregulation of the protein are represented as (↑) and (↓), respectively, based on the ratio that was obtained. The data were compared individually with one another using PEAKS Q. N number is 3 for all controls and samples, whereas, the *p*-value was set at 0.05 significance level.

**Table 2:** Forward and reverse primers that were used to study gene expression changes via qPCR.

Target Gene	Sequence	References
TNF-α	Forward: 5′-CCAGGCAGTCAGATCATCTTCTC-3′	[130]
	Reverse: 5′-AGCTTGAGGGTTTGCTACAACAT-3′	
NF-κB	Forward: 5′-GACGAGAACGGAGACACA-3′	Designed with NCBI Primer Blast
	Reverse: 5′-TGGTTGGTAGGTTGACAAC-3′	
COX-2	Forward 5′-TGCGCCTTTTCAAGGATGGA-3′	Designed with NCBI Primer Blast
	Reverse 5′-CCCCACAGCAAACCGTAGAT-3′	
MMP-1	Forward 5′-GGGAGATCATCGGGACAACTC-3′	[131]
	Reverse 5′-TGAGCATCCCCTCCAATACC-3′	
Cyclin D1	Forward 5′-TGCGCTGCTACCGTTGACT-3′	[132]
	Reverse 5′-AGCGATGTGAATATTTCCAAACC-3′	
Bax	Forward 5′-GTCGCCCTTTTCTACTTTGCCAG-3′	[126]
	Reverse 5′-TCCAGCCCAACAGCCGCTCC-3′	
18S ribosomal RNA	Forward: 5′-GGCCCTGTAATTGGAATGAGTC-3′	[133]
	Reverse: 5′-CCAAGATCCAACTACGAGCTT-3′	

## Data Availability

The data used to support the findings of this study are available from the corresponding author upon request.

## Supplementary Materials

The following are available online. Table S1: Analysis of differentially expressed protein in UVB control cells and those irradiated but treated with *S. macrophylla* extract and fractions via LC-MS/MS.
