# Supplementary material for: Counteracting the Ramifications of UVB Irradiation and Photoaging with Swietenia macrophylla King Seed
Source: Molecules. 2021 Apr 1;26(7):2000. doi: 10.3390/molecules26072000 (PMC8037697; doi:10.3390/molecules26072000)
Supplement: Supplementary file 1 [file molecules-26-02000-s001.pdf]

Supplementary Materials

Table S1. Analysis of differentially expressed protein in UVB control cells and those irradiated but treated with *S. macrophylla* extract and fractions via LC-MS/MS.

No.	Protein Name	Uniprot ID	Group Comparison	Accession	Significance	No. of Peptides	No. of Unique Peptides	Sample Profile (Ratio)	Average Mass
1	Chaperonin containing TCP1 subunit 2 β isoform CRA_b (CCT-β)	V9HW96	Controls = Non-UVB : UVB	tr|V9HW96|V9HW96_HUMAN	18.24	5	5	1.00:0.49	57488
2			Sample = UVB : SMHF + UVB	tr|V9HW96|V9HW96_HUMAN	19.02	4	4	1.00:1.41	57488
3	T-complex protein 1 subunit delta (CCT-δ)	A8K3C3	Controls = Non-UVB : UVB	tr|Q05CK9|Q05CK9_HUMAN	14.1	1	1	1.00:0.48	50651
4			Sample = UVB : SMHF + UVB	tr|A8K3C3|A8K3C3_HUMAN	39.86	3	3	1.00:1.71	57952
5			Sample = UVB : SMWF + UVB	tr|A8K3C3|A8K3C3_HUMAN	57.57	1	1	1.00:0.52	57952
6	T-complex protein 1 subunit gamma (CCT-γ)	B3KX11	Sample = UVB : SMHF + UVB	tr|B3KX11|B3KX11_HUMAN	20.6	2	2	1.00:1.58	57946
7	T-complex protein 1 subunit eta (CCT-η)	Q99832	Sample = UVB : SMHF + UVB	Q99832|TCPH_HUMAN	55.72	3	3	1.00:1.92	59367
8	T-complex protein 1 subunit epsilon (CCT-ε)	P48643	Sample = UVB : SMHF + UVB	P48643|TCPE_HUMAN	20.61	1	1	1.00:2.39	59671
9	Ribosomal protein L12 variant	Q59FI9	Controls = Non-UVB : UVB	tr|Q59FI9|Q59FI9_HUMAN	57.92	1	1	1.00:0.34	21483
10	Ribosomal protein S8	Q9BS10	Sample = UVB : SMHF + UVB	tr|Q9BS10|Q9BS10_HUMAN	54.63	1	1	1.00:1.90	9283
11	40S ribosomal protein SA	C9J9K3	Sample = UVB : SMHF + UVB	tr|C9J9K3|C9J9K3_HUMAN	51.73	4	4	1.00:1.87	29405

12	40S ribosomal protein S5	M0QZN2	Controls = Non-UVB : UVB	tr|M0QZN2|M0QZN2_HUMAN	38.51	1	1	1.00:0.32	14763
13			Sample = UVB : SMWF + UVB	tr|M0QZN2|M0QZN2_HUMAN	15.93	1	1	1.00:0.32	14763
14	40S ribosomal protein S7	P62081	Controls = Non-UVB : UVB	P62081|RS7_HUMAN	17.77	2	2	1.00:0.45	22127
15			Sample = UVB : SMWF + UVB	P62081|RS7_HUMAN	200	1	1	1.00:0.29	22127
16	40S ribosomal protein S10	P46783	Controls = Non-UVB : UVB	P46783|RS10_HUMAN	37.75	1	1	1.00:0.33	18898
17			Sample = UVB : SMWF + UVB	P46783|RS10_HUMAN	200	1	1	1.00:0.31	18898
18	40S ribosomal protein S16	M0R210	Controls = Non-UVB : UVB	tr|M0R210|M0R210_HUMAN	47.51	2	2	1.00:0.36	14419
19		M0R3H0	Sample = UVB : SMWF + UVB	tr|M0R3H0|M0R3H0_HUMAN	59.56	2	2	1.00:0.51	11075
20	40S ribosomal protein S25	P62851	Controls = Non-UVB : UVB	P62851|RS25_HUMAN	22.34	2	2	1.00:0.48	13742
21			Sample = UVB : SMWF + UVB	P62851|RS25_HUMAN	62.68	1	1	1.00:0.50	13742
22	60S ribosomal protein L6	Q8N5Z7	Controls = Non-UVB : UVB	tr|Q8N5Z7|Q8N5Z7_HUMAN	55.27	2	2	1.00:0.31	32726

<table>
<tr><th></th><th></th><th></th><th></th><th></th><th></th><th></th><th></th><th></th><th></th></tr>
<tr><td>23</td><td>60S ribosomal protein L18</td><td>F8VUA6</td><td>Controls = Non-UVB : UVB</td><td>tr|F8VUA6|F8VUA6_HUMAN</td><td>16.78</td><td>1</td><td>1</td><td>1.00:0.51</td><td>14529</td></tr>
<tr><td>24</td><td></td><td></td><td>Sample = UVB : SMCE + UVB</td><td>tr|F8VUA6|F8VUA6_HUMAN</td><td>15.02</td><td>1</td><td>1</td><td>1.00:0.22</td><td>14529</td></tr>
<tr><td>25</td><td></td><td></td><td>Sample = UVB : SMHF + UVB</td><td>tr|F8VUA6|F8VUA6_HUMAN</td><td>22.98</td><td>1</td><td>1</td><td>1.00:0.68</td><td>14529</td></tr>
<tr><td>26</td><td></td><td></td><td>Sample = UVB : SMWF + UVB</td><td>tr|F8VUA6|F8VUA6_HUMAN</td><td>83.69</td><td>1</td><td>1</td><td>1.00:0.44</td><td>14529</td></tr>
<tr><td>27</td><td>60S ribosomal protein L22</td><td>K7EJT5</td><td>Controls = Non-UVB : UVB</td><td>tr|K7EJT5|K7EJT5_HUMAN</td><td>51.28</td><td>2</td><td>2</td><td>1.00:0.37</td><td>5083</td></tr>
<tr><td>28</td><td></td><td></td><td>Sample = UVB : SMHF + UVB</td><td>tr|K7EJT5|K7EJT5_HUMAN</td><td>39.81</td><td>1</td><td>1</td><td>1.00:1.71</td><td>5083</td></tr>
<tr><td>29</td><td>60S ribosomal protein L24</td><td>C9JXB8</td><td>Controls = Non-UVB : UVB</td><td>tr|C9JXB8|C9JXB8_HUMAN</td><td>18.7</td><td>1</td><td>1</td><td>1.00:0.48</td><td>14369</td></tr>
<tr><td>30</td><td>60S ribosomal protein L29</td><td>A0A024R326</td><td>Controls = Non-UVB : UVB</td><td>tr|A0A024R326|A0A024R326_HUMAN</td><td>15.98</td><td>1</td><td>1</td><td>1.00:0.50</td><td>17553</td></tr>
<tr><td>31</td><td></td><td></td><td>Sample = UVB : SMWF + UVB</td><td>tr|A0A024R326|A0A024R326_HUMAN</td><td>107.42</td><td>1</td><td>1</td><td>1.00:0.39</td><td>17553</td></tr>
<tr><td>32</td><td>60s acidic ribosomal protein P0</td><td>Q53HW2</td><td>Sample = UVB : SMWF + UVB</td><td>tr|Q53HW2|Q53HW2_HUMAN</td><td>22.82</td><td>4</td><td>4</td><td>1.00:0.40</td><td>34302</td></tr>
<tr><td>33</td><td>60s acidic ribosomal protein P2</td><td>P05387</td><td>Sample = UVB : SMHF + UVB</td><td>P05387|RLA2_HUMAN</td><td>17.44</td><td>3</td><td>3</td><td>1.00:1.39</td><td>11665</td></tr>
<tr><td>34</td><td>Albumin</td><td>F6KPG5</td><td>Controls = Non-UVB : UVB</td><td>tr|F6KPG5|F6KPG5_HUMAN</td><td>44.37</td><td>5</td><td>5</td><td>1.00:2.66</td><td>66531</td></tr>
<tr><td>35</td><td></td><td></td><td>Sample = UVB : SMWF + UVB</td><td>tr|F6KPG5|F6KPG5_HUMAN</td><td>96.58</td><td>5</td><td>5</td><td>1.00:0.41</td><td>66531</td></tr>
<tr><td>36</td><td>chloride intracellular channel</td><td>A0A1U9X8Y4</td><td>Sample = UVB : SMWF + UVB</td><td>tr|A0A1U9X8Y4|A0A1U9X8Y4_HUMAN</td><td>30.98</td><td>4</td><td>4</td><td>1.00:0.63</td><td>26794</td></tr>
<tr><td>37</td><td>chloride intracellular channel 1 (CLIC1)</td><td>O00299</td><td>Controls = Non-UVB : UVB</td><td>O00299|CLIC1_HUMAN</td><td>16.55</td><td>4</td><td>4</td><td>1.00:0.56</td><td>26923</td></tr>
<tr><td>38</td><td></td><td></td><td>Sample = UVB : SMHF + UVB</td><td>O00299|CLIC1_HUMAN</td><td>28.31</td><td>5</td><td>5</td><td>1.00:1.55</td><td>26923</td></tr>
</table>

39			Controls = Non-UVB : UVB	tr∣V9HWI5∣V9HWI5_HUMAN	21.63	8	8	1.00:0.61	18502
40	Cofilin 1	V9HWI5	Sample = UVB : SMHF + UVB	tr∣V9HWI5∣V9HWI5_HUMAN	17.19	8	8	1.00:1.38	18502
41			Sample = UVB : SMWF + UVB	tr∣V9HWI5∣V9HWI5_HUMAN	30.35	8	8	1.00:0.63	18502
42	Elongation factor 1-alpha	Q6IPN6	Controls = Non-UVB : UVB	tr∣Q6IPN6∣Q6IPN6_HUMAN	38.38	3	3	1.00:0.36	50123
43		Q53HM9	Sample = UVB : SMHF + UVB	tr∣Q53HM9∣Q53HM9_HUMAN	52.68	7	7	1.00:1.88	50142
44		A0A087X1X7	Controls = Non-UVB : UVB	tr∣A0A087X1X7∣A0A087X1X7_HUMAN	66.57	1	1	1.00:0.33	69283
45	Elongation factor 1-delta		Sample = UVB : SMHF + UVB	tr∣E9PN91∣E9PN91_HUMAN	25.14	1	1	1.00:1.51	11616
46		E9PN91	Sample = UVB : SMEAF + UVB	tr∣E9PN91∣E9PN91_HUMAN	23.86	1	1	1.00:1.49	11616
47			Sample = UVB : SMWF + UVB	tr∣E9PN91∣E9PN91_HUMAN	24.9	1	1	1.00:0.66	11616
48	Eukaryotic translation elongation factor 2 (eEF2) (Epididymis secretory sperm binding protein)		Controls = Non-UVB : UVB	tr∣A0A384N6H1∣A0A384N6H1_HUMAN	20.93	23	23	1.00:0.55	95338
49		A0A384N6H1	Sample = UVB : SMHF + UVB	tr∣A0A384N6H1∣A0A384N6H1_HUMAN	36.41	22	22	1.00:1.67	95338
50			Sample = UVB : SMWF + UVB	tr∣A0A384N6H1∣A0A384N6H1_HUMAN	101.17	20	20	1.00:0.41	95338

51	Glycine-tRNA ligase	P41250	Controls = Non-UVB : UVB	P41250|GARS_HUMAN	28.56	2	2	1.00:0.28	83166
52			Sample = UVB : SMHF + UVB	P41250|GARS_HUMAN	18.66	1	1	1.00:7.22	83166
53	HSP-10 kDa (Chaperonin 10) (Epididymis secretory sperm binding protein)	A0A384N6A4	Sample = UVB : SMHF + UVB	tr|A0A384N6A4|A0A384N6A4_HUMAN	23.46	6	6	1.00:1.48	10932
54			Sample = UVB : SMWF + UVB	tr|A0A384N6A4|A0A384N6A4_HUMAN	54.42	5	5	1.00:0.53	10932
55	HSP-60 kDa mitochondrial	P10809	Sample = UVB : SMWF + UVB	P10809|CH60_HUMAN	56.7	26	26	1.00:0.52	61055
56	HSP-70 kDa protein 1A variant	Q59EJ3	Sample = UVB : SMWF + UVB	tr|Q59EJ3|Q59EJ3_HUMAN	14.31	7	7	1.00:0.54	77496
57	HSP-70 kDa protein 4	Q59GF8	Sample = UVB : SMHF + UVB	tr|Q59GF8|Q59GF8_HUMAN	15.99	1	1	1.00:1.36	88005
58	HSP-70 family protein 5 (Epididymis secretory sperm binding protein Li 89n) (78 kDa glucose-regulated protein)	V9HWB4	Sample = UVB : SMWF + UVB	tr|V9HWB4|V9HWB4_HUMAN	51.57	19	19	1.00:0.54	72333
59	HSP-70 kDa protein 8, isoform CRA_a (Epididymis luminal protein 33)	V9HW22	Controls = Non-UVB : UVB	tr|V9HW22|V9HW22_HUMAN	29.01	21	21	1.00:0.52	70898
60			Sample = UVB : SMHF + UVB	tr|V9HW22|V9HW22_HUMAN	24.34	17	17	1.00:1.50	70898
61	HSP-70 kDa protein 9 (75 kDa glucose-regulated protein)	B7Z4V2	Sample = UVB : SMHF + UVB	tr|B7Z4V2|B7Z4V2_HUMAN	15.17	3	3	1.00:1.35	72401
62	HSP 90α	P07900	Controls = Non-UVB : UVB	P07900|HS90A_HUMAN	17.64	12	12	1.00:0.60	84660
63			Sample = UVB : SMHF + UVB	P07900|HS90A_HUMAN	25.18	14	14	1.00:1.51	84660
64			Sample = UVB : SMWF + UVB	P07900|HS90A_HUMAN	59.44	12	12	1.00:0.51	84660

65	Gluthathione S-transferase (GST)-pi (Epididymis secretory protein Li 22)	*V9HWE9*	Controls = Non-UVB : UVB	tr∣V9HWE9∣V9HWE9_HUMAN	25	8	8	1.00:0.63	23356
66			Sample = UVB : SMHF + UVB	tr∣V9HWE9∣V9HWE9_HUMAN	21.62	10	10	1.00:1.45	23356
67			Sample = UVB : SMWF + UVB	tr∣V9HWE9∣V9HWE9_HUMAN	39.86	9	9	1.00:0.58	23356
68	Poly (RC) binding protein 1 (Epididymis secretory protein Li 85)	Q53SS8	Controls = Non-UVB : UVB	tr∣Q53SS8∣Q53SS8_HUMAN	38.97	1	1	1.00:0.32	37498
69			Sample = UVB : SMHF + UVB	tr∣Q53SS8∣Q53SS8_HUMAN	70.34	1	1	1.00:2.10	37498
70			Sample = UVB : SMWF + UVB	tr∣Q53SS8∣Q53SS8_HUMAN	20.78	1	1	1.00:0.56	37498
71	Histone H2A type 1-A	Q96QV6	Sample = UVB : SMEAF + UVB	Q96QV6∣H2A1A_HUMAN	18.55	2	2	1.00:1.41	14233
72	Histone H2A type 2-B	Q8IUE6	Controls = Non-UVB : UVB	Q8IUE6∣H2A2B_HUMAN	44.08	1	1	1.00:2.75	13995
73			Sample = UVB : SMHF + UVB	Q8IUE6∣H2A2B_HUMAN	36.1	2	2	1.00:1.66	13995
74			Sample = UVB : SMWF + UVB	Q8IUE6∣H2A2B_HUMAN	44.36	1	1	1.00:0.56	13995
75	Histone H2B	A8K9J7	Controls = Non-UVB : UVB	tr∣A8K9J7∣A8K9J7_HUMAN	41.67	3	3	1.00:2.86	13996
76		B4DR52	Sample = UVB : SMWF + UVB	tr∣B4DR52∣B4DR52_HUMAN	106.12	3	3	1.00:0.40	18041
77	Histone H1.2	P16403	Sample = UVB : SMHF + UVB	P16403∣H12_HUMAN	37.88	2	2	1.00:1.69	21365
78			Sample = UVB : SMEAF + UVB	P16403∣H12_HUMAN	15.93	2	2	1.00:1.41	21365
79			Sample = UVB : SMWF + UVB	P16403∣H12_HUMAN	32.49	2	2	1.00:0.62	21365

80	Histone H1.5	P16401	Sample = UVB : SMHF + UVB	P16401|H15_HUMAN	62.86	1	1	1.00:2.01	22580
81	Histone H4	B2R4R0	Sample = UVB : SMWF + UVB	tr|B2R4R0|B2R4R0_HUMAN	60.9	4	4	1.00:0.50	11367
82	Myosin light polypeptide 6	F8W1R7	Controls = Non-UVB : UVB	tr|F8W1R7|F8W1R7_HUMAN	33.23	2	2	1.00:0.43	16290
83	Myosin 9	P35579	Controls = Non-UVB : UVB	P35579|MYH9_HUMAN	20.81	6	6	1.00:0.48	226530
84			Sample = UVB : SMWF + UVB	P35579|MYH9_HUMAN	55.52	3	3	1.00:0.52	226530
85	Nucleophosmin (Nucleolar phosphoprotein B23 numatrin) isoform CRA_f	A0A0S2Z4G7	Controls = Non-UVB : UVB	tr|A0A0S2Z4G7|A0A0S2Z4G7_HUMAN	19.08	6	6	1.00:0.71	29465
86			Sample = UVB : SMHF + UVB	tr|A0A0S2Z4G7|A0A0S2Z4G7_HUMAN	14.56	7	7	1.00:1.34	29465
87			Sample = UVB : SMWF + UVB	tr|A0A0S2Z4G7|A0A0S2Z4G7_HUMAN	89.01	7	7	1.00:0.43	29465
88	Peptidyl-prolyl cis-trans isomerase	V9HWF5	Controls = Non-UVB : UVB	tr|V9HWF5|V9HWF5_HUMAN	44.93	13	13	1.00:0.56	18012
89			Sample = UVB : SMWF + UVB	tr|V9HWF5|V9HWF5_HUMAN	89.29	13	13	1.00:0.43	18012
90	Peroxiredoxin (RPDX)-1	A0A384NPQ2	Controls = Non-UVB : UVB	tr|A0A384NPQ2|A0A384NPQ2_HUMAN	26.93	8	8	1.00:0.57	22110
91		A0A0A0MSI0	Sample = UVB : SMHF + UVB	tr|A0A0A0MSI0|A0A0A0MSI0_HUMAN	36.67	7	7	1.00:1.67	18976
92			Sample = UVB : SMWF + UVB	tr|A0A0A0MSI0|A0A0A0MSI0_HUMAN	90.09	7	7	1.00:0.43	18976

93	PRDX-2	B4DF70	Sample = UVB : SMWF + UVB	tr|B4DF70|B4DF70_HUMAN	14.43	3	3	1.00:0.51	20107
94	PRDX-3 (Thioredoxin-dependent peroxide reductase mitochondrial)	P30048	Sample = UVB : SMHF + UVB	P30048|PRDX3_HUMAN	40.66	2	2	1.00:0.58	27693
95			Sample = UVB : SMEAF + UVB	P30048|PRDX3_HUMAN	16.64	2	2	1.00:0.73	27693
96	PRDX-6	P30041	Controls = Non-UVB : UVB	P30041|PRDX6_HUMAN	22.55	9	9	1.00:0.58	25035
97			Sample = UVB : SMWF + UVB	P30041|PRDX6_HUMAN	95.57	7	7	1.00:0.42	25035
98	Phosphoglycerate kinase 1	P00558	Controls = Non-UVB : UVB	P00558|PGK1_HUMAN	17.22	9	9	1.00:0.61	44615
99			Sample = UVB : SMHF + UVB	P00558|PGK1_HUMAN	30.3	9	9	1.00:1.58	44615
100			Sample = UVB : SMWF + UVB	P00558|PGK1_HUMAN	61.3	5	5	1.00:0.50	44615
101	Phosphoserine aminotransferase	Q9Y617	Controls = Non-UVB : UVB	Q9Y617|SERC_HUMAN	15.37	5	5	1.00:0.56	40423
102			Sample = UVB : SMHF + UVB	Q9Y617|SERC_HUMAN	22.11	5	5	1.00:1.46	40423
103			Sample = UVB : SMWF + UVB	Q9Y617|SERC_HUMAN	109.97	3	3	1.00:0.39	40423
104	Profilin-1	P07737	Controls = Non-UVB : UVB	P07737|PROF1_HUMAN	30.82	7	7	1.00:0.63	15054
105			Sample = UVB : SMHF + UVB	P07737|PROF1_HUMAN	19.13	7	7	1.00:1.42	15054
106			Sample = UVB : SMWF + UVB	P07737|PROF1_HUMAN	45.17	7	7	1.00:0.56	15054

107	Protein disulfide-isomerase (PDI)	A0A024R8S5	Sample = UVB : SMHF + UVB	tr|A0A024R8S5|A0A024R8S5_HUMAN	26.06	6	6	1.00:1.52	57116
108			Sample = UVB : SMWF + UVB	tr|A0A024R8S5|A0A024R8S5_HUMAN	29.85	6	6	1.00:0.63	57116
109	PDI-A3	P30101	Sample = UVB : SMHF + UVB	P30101|PDIA3_HUMAN	35.84	20	20	1.00:1.66	56782
110			Sample = UVB : SMEAF + UVB	P30101|PDIA3_HUMAN	13.15	18	18	1.00:0.76	56782
111	PDI-A4	P13667	Sample = UVB : SMHF + UVB	P13667|PDIA4_HUMAN	45.66	2	2	1.00:2.30	72933
112			Sample = UVB : SMWF + UVB	P13667|PDIA4_HUMAN	35.87	1	1	1.00:0.60	72933
113	PDI-A6 (Endoplasmic reticulum protein 5)	Q15084	Controls = Non-UVB : UVB	Q15084|PDIA6_HUMAN	15.59	2	2	1.00:1.74	48121
114			Sample = UVB : SMWF + UVB	Q15084|PDIA6_HUMAN	90.53	4	4	1.00:0.43	48121
115	Protein S100	A0A590UJ49	Controls = Non-UVB : UVB	tr|A0A590UJ49|A0A590UJ49_HUMAN	21.87	3	3	1.00:0.30	10986
116		B2R5H0	Sample = UVB : SMHF + UVB	tr|B2R5H0|B2R5H0_HUMAN	22.31	5	5	1.00:1.47	11724
117			Sample = UVB : SMWF + UVB	tr|B2R5H0|B2R5H0_HUMAN	146.54	3	3	1.00:0.33	11724
118	Protein-arginine deiminase type-1	Q9ULC6	Controls = Non-UVB : UVB	Q9ULC6|PADI1_HUMAN	30.5	1	1	1.00:15.79	74666
119			Sample = UVB : SMWF + UVB	Q9ULC6|PADI1_HUMAN	36.48	1	1	1.00:0.21	74666

120			Controls = Non-UVB : UVB	P14618|KPYM_HUMAN	14.55	29	29	1.00:0.67	57937
121	Pyruvate kinase PKM	P14618	Sample = UVB : SMHF + UVB	P14618|KPYM_HUMAN	13.96	26	26	1.00:1.33	57937
122			Sample = UVB : SMWF + UVB	P14618|KPYM_HUMAN	70.32	22	22	1.00:0.48	57937
123	Receptor of activated protein C kinase 1	P63244	Controls = Non-UVB : UVB	P63244|RACK1_HUMAN	52.92	1	1	1.00:0.22	35077
124		D6RF23	Sample = UVB : SMWF + UVB	tr|D6RF23|D6RF23_HUMAN	50.25	1	1	1.00:9.32	11247
125	Signal recognition particle 14 kDa protein (SRP-14)	P37108	Controls = Non-UVB : UVB	P37108|SRP14_HUMAN	27.41	1	1	1.00:0.33	14570
126	SYNCRIP protein	Q05CK9	Controls = Non-UVB : UVB	tr|Q05CK9|Q05CK9_HUMAN	14.1	1	1	1.00:0.48	50651
127	Transketolase	B3KSI4	Controls = Non-UVB : UVB	tr|B3KSI4|B3KSI4_HUMAN	17.58	4	4	1.00:0.52	58982
128	D-3-phosphoglycerate dehydrogenase	B3KSC3	Controls = Non-UVB : UVB	tr|B3KSC3|B3KSC3_HUMAN	15.42	2	2	1.00:0.50	53013
129	Fumarate hydratase (Epididymis secretory sperm binding protein)	A0A0S2Z4C3	Sample = UVB : SMCE + UVB	tr|A0A0S2Z4C3|A0A0S2Z4C3_HUMAN	13.45	2	2	1.00:2.02	54637
130			Sample = UVB : SMHF + UVB	tr|A0A0S2Z4C3|A0A0S2Z4C3_HUMAN	77.24	2	2	1.00:2.43	54637
131	Annexin A1	Q5TZZ9	Sample = UVB : SMHF + UVB	tr|Q5TZZ9|Q5TZZ9_HUMAN	22.03	20	20	1.00:1.46	38714
132			Sample = UVB : SMWF + UVB	tr|Q5TZZ9|Q5TZZ9_HUMAN	87.99	20	20	1.00:0.43	38714

133	Annexin A2	A0A024R5Z7	Sample = UVB : SMWF + UVB	tr|A0A024R5Z7|A0A024R5Z7_HUMAN	51.92	15	15	1.00:0.46	38604
134	Annexin A3	P12429	Sample = UVB : SMCE + UVB	P12429|ANXA3_HUMAN	13.3	3	3	1.00:0.63	36375
135			Sample = UVB : SMEAF + UVB	P12429|ANXA3_HUMAN	55.68	3	3	1.00:0.52	36375
136			Sample = UVB : SMWF + UVB	P12429|ANXA3_HUMAN	96.02	2	2	1.00:0.42	36375
137	Annexin A5	P08758	Sample = UVB : SMWF + UVB	P08758|ANXA5_HUMAN	92.36	8	8	1.00:0.42	35937
138	Filamin A	Q60FE6	Sample = UVB : SMHF + UVB	tr|Q60FE6|Q60FE6_HUMAN	27.19	1	1	1.00:1.54	277503
139			Sample = UVB : SMWF + UVB	tr|Q60FE6|Q60FE6_HUMAN	126.49	1	1	1.00:0.36	277503
140	Filamin B β (Actin binding protein 278) isoform CRA_a	A0A024R321	Sample = UVB : SMCE + UVB	tr|A0A024R321|A0A024R321_HUMAN	13.25	1	1	1.00:0.44	280488
141	3-phosphoglycerate dehydrogenase	Q9UMY2	Sample = UVB : SMHF + UVB	tr|Q9UMY2|Q9UMY2_HUMAN	85.14	2	2	1.00:2.27	43082
142		Q9UMY3	Sample = UVB : SMWF + UVB	tr|Q9UMY3|Q9UMY3_HUMAN	34.43	1	1	1.00:0.26	24159
143	14-3-3 protein α/β	P31946	Sample = UVB : SMWF + UVB	P31946|1433B_HUMAN	36.85	4	4	1.00:0.60	28082
144	14-3-3 protein σ	P31947	Sample = UVB : SMHF + UVB	P31947|1433S_HUMAN	59.67	12	12	1.00:1.97	27774
145			Sample = UVB : SMWF + UVB	P31947|1433S_HUMAN	71.08	12	12	1.00:0.47	27774
146	14-3-3 protein γ	P61981	Sample = UVB : SMWF + UVB	P61981|1433G_HUMAN	200	1	1	1.00:0.18	28303
147	14-3-3 protein ε	P62258	Sample = UVB : SMWF + UVB	P62258|1433E_HUMAN	116.41	7	7	1.00:0.38	29174

148	Ubiquitin-activating enzyme E1 (Testicular secretory protein Li 63)	A0A024R1A3	Sample = UVB : SMHF + UVB	tr∣A0A024R1A3∣A0A024R1A3_HUMAN	49.55	5	5	1.00:1.84	117849
149	Neuroblast differentiation-associated protein AHNAK (AHNAK) (Desmoyokin)	Q09666	Sample = UVB : SMHF + UVB	Q09666∣AHNK_HUMAN	48.61	4	4	1.00:0.55	629114
150	Keratin type I cytoskeletal 14	P02533	Sample = UVB : SMHF + UVB	P02533∣K1C14_HUMAN	41.95	1	1	1.00:1.86	51562
151			Sample = UVB : SMEAF + UVB	P02533∣K1C14_HUMAN	13.67	3	3	1.00:1.88	51562
152	Keratin type II cytoskeletal 8	P05787	Sample = UVB : SMHF + UVB	P05787∣K2C8_HUMAN	23.35	7	7	1.00:1.48	53704
153			Sample = UVB : SMWF + UVB	P05787∣K2C8_HUMAN	36.47	6	6	1.00:0.52	53704
154	Heterogeneous nuclear ribonucleoprotein (hnRP) D0	D6RF44	Sample = UVB : SMHF + UVB	tr∣D6RF44∣D6RF44_HUMAN	41.78	2	2	1.00:1.74	12553
155	hnRP K	B4DFF1	Sample = UVB : SMHF + UVB	tr∣B4DFF1∣B4DFF1_HUMAN	27.06	3	3	1.00:1.54	50715
156		B4DUQ1	Sample = UVB : SMWF + UVB	tr∣B4DUQ1∣B4DUQ1_HUMAN	59.78	3	3	1.00:0.51	48511
157	Adenylyl cyclase-associated protein (CAP1)	B4DI38	Sample = UVB : SMHF + UVB	tr∣B4DI38∣B4DI38_HUMAN	27.03	4	4	1.00:1.54	49080
158			Sample = UVB : SMWF + UVB	tr∣B4DI38∣B4DI38_HUMAN	113.48	2	2	1.00:0.38	49080

159	Reticulon-4	Q6IPN0	Sample = UVB : SMHF + UVB	tr|Q6IPN0|Q6IPN0_HUMAN	38.91	1	1	1.00:1.70	36918
160		Q9NQC3	Sample = UVB : SMWF + UVB	Q9NQC3|RTN4_HUMAN	28.35	1	1	1.00:0.64	129931
161	Malate dehydrogenase cytoplasmic	P40925	Sample = UVB : SMHF + UVB	P40925|MDHC_HUMAN	38.86	3	3	1.00:1.70	36426
162			Sample = UVB : SMWF + UVB	P40925|MDHC_HUMAN	17.61	1	1	1.00:0.72	36426
163	Transketolase	A0A0B4J1R6	Sample = UVB : SMHF + UVB	tr|A0A0B4J1R6|A0A0B4J1R6_HUMAN	33.06	4	4	1.00:1.62	49910
164			Sample = UVB : SMWF + UVB	tr|A0A0B4J1R6|A0A0B4J1R6_HUMAN	140.62	3	3	1.00:0.34	49910
165	Glucose-6-phosphate isomerase	P06744	Sample = UVB : SMHF + UVB	P06744|G6PI_HUMAN	32.62	6	6	1.00:1.61	63147
166			Sample = UVB : SMWF + UVB	P06744|G6PI_HUMAN	129.15	3	3	1.00:0.36	63147
167	Proteasome subunit alpha type	Q53GF5	Sample = UVB : SMHF + UVB	tr|Q53GF5|Q53GF5_HUMAN	13.19	1	1	1.00:1.45	25841
168		H0YLC2	Sample = UVB : SMWF + UVB	tr|H0YLC2|H0YLC2_HUMAN	66.14	2	2	1.00:0.32	19654
169	Proteasome activator complex subunit 2	Q86SZ7	Sample = UVB : SMHF + UVB	tr|Q86SZ7|Q86SZ7_HUMAN	24.42	3	3	1.00:1.63	27402
170	Proteasome subunit β type-2	P49721	Sample = UVB : SMWF + UVB	P49721|PSB2_HUMAN	14.02	1	1	1.00:0.42	22836
171	Proteasome subunit β type-3	A0A087WXQ8	Sample = UVB : SMHF + UVB	tr|A0A087WXQ8|A0A087WXQ8_HUMAN	30.79	1	1	1.00:1.59	12082
172	Glyceraldehyde-3-phosphate dehydrogenase (GAPDH)	P04406	Sample = UVB : SMHF + UVB	P04406|G3P_HUMAN	29.22	14	14	1.00:1.57	36053
173			Sample = UVB : SMWF + UVB	P04406|G3P_HUMAN	71.64	13	13	1.00:0.47	36053

174	Proliferating cell nuclear antigen (PCNA)	P12004	Sample = UVB : SMHF + UVB	P12004 ǀ PCNA_HUMAN	28.29	4	4	1.00:1.55	28769
175		Q6FHF5	Sample = UVB : SMWF + UVB	tr ǀ Q6FHF5 ǀ Q6FHF5_HUMAN	52.21	1	1	1.00:0.53	28706
176	Exportin-2	P55060	Sample = UVB : SMHF + UVB	P55060 ǀ XPO2_HUMAN	26.67	3	3	1.00:1.62	110417
177			Sample = UVB : SMEAF + UVB	P55060 ǀ XPO2_HUMAN	14.11	3	3	1.00:1.33	110417
178	Serpin peptidase inhibitor clade B (Ovalbumin) member 5 isoform CRA_b (SERPINB5)	A0A024R2B6	Sample = UVB : SMHF + UVB	tr ǀ A0A024R2B6 ǀ A0A024R2B6_HUMAN	25.77	7	7	1.00:1.52	42100
179			Sample = UVB : SMWF + UVB	tr ǀ A0A024R2B6 ǀ A0A024R2B6_HUMAN	58.84	6	6	1.00:0.51	42100
180	Collagen-binding protein (Serpin H1)	B4DN87	Sample = UVB : SMHF + UVB	tr ǀ B4DN87 ǀ B4DN87_HUMAN	46.74	4	4	1.00:1.80	44204
181	Ezrin	Q6NUR7	Sample = UVB : SMHF + UVB	tr ǀ Q6NUR7 ǀ Q6NUR7_HUMAN	25.09	2	2	1.00:1.51	69242
182			Sample = UVB : SMWF + UVB	tr ǀ Q6NUR7 ǀ Q6NUR7_HUMAN	200	1	1	1.00:0.19	69242
183	Tubulin beta chain	A0A384NYT8	Sample = UVB : SMHF + UVB	tr ǀ A0A384NYT8 ǀ A0A384NYT8_HUMAN	22.95	3	3	1.00:1.47	49469
184			Sample = UVB : SMWF + UVB	tr ǀ A0A384NYT8 ǀ A0A384NYT8_HUMAN	65.21	3	3	1.00:0.49	49469
185	Thioredoxin	P10599	Sample = UVB : SMHF + UVB	P10599 ǀ THIO_HUMAN	17.97	6	6	1.00:1.40	11737
186			Sample = UVB : SMWF + UVB	P10599 ǀ THIO_HUMAN	50.68	5	5	1.00:0.54	11737

187	Thioredoxin domain-containing protein 17 (TXNDC17) (Testicular tissue protein Li 214)	A0A140VJY7	Sample = UVB : SMHF + UVB	tr|A0A140VJY7|A0A140VJY7_HUMAN	22.76	2	2	1.00:1.47	13941
188	Alpha-enolase	P06733	Sample = UVB : SMHF + UVB	P06733|ENOA_HUMAN	21.88	19	19	1.00:1.46	47169
189			Sample = UVB : SMWF + UVB	P06733|ENOA_HUMAN	77.84	20	20	1.00:0.46	47169
190	Triosephosphate isomerase	V9HWK1	Sample = UVB : SMHF + UVB	tr|V9HWK1|V9HWK1_HUMAN	21.24	12	12	1.00:1.45	26669
191			Sample = UVB : SMWF + UVB	tr|V9HWK1|V9HWK1_HUMAN	84.41	5	5	1.00:0.44	26669
192	Alpha-actinin-1	P12814	Sample = UVB : SMHF + UVB	P12814|ACTN1_HUMAN	20.99	2	2	1.00:1.44	103058
193	Calreticulin variant	Q53G71	Sample = UVB : SMHF + UVB	tr|Q53G71|Q53G71_HUMAN	20.7	8	8	1.00:1.44	46919
194			Sample = UVB : SMWF + UVB	tr|Q53G71|Q53G71_HUMAN	96.13	3	3	1.00:0.42	46919
195	L-lactate dehydrogenase A	V9HWB9	Sample = UVB : SMHF + UVB	tr|V9HWB9|V9HWB9_HUMAN	16.74	16	16	1.00:1.38	36689
196			Sample = UVB : SMWF + UVB	tr|V9HWB9|V9HWB9_HUMAN	135.57	13	13	1.00:0.35	36689
197	L-lactate dehydrogenase B	Q5U077	Sample = UVB : SMHF + UVB	tr|Q5U077|Q5U077_HUMAN	17.73	10	10	1.00:1.39	36639
198			Sample = UVB : SMWF + UVB	tr|Q5U077|Q5U077_HUMAN	93.01	9	9	1.00:0.42	36639
199	ATP synthase subunit α, mitochondrial	V9HW26	Sample = UVB : SMWF + UVB	tr|V9HW26|V9HW26_HUMAN	17.06	8	8	1.00:0.44	59751

200	ATP synthase subunit β, mitochondrial	P06576	Sample = UVB : SMHF + UVB	P06576|ATPB_HUMAN	16.57	17	17	1.00:1.37	56560
201			Sample = UVB : SMWF + UVB	P06576|ATPB_HUMAN	37.56	11	11	1.00:0.59	56560
202	Endoplasmin	P14625	Sample = UVB : SMHF + UVB	P14625|ENPL_HUMAN	15.57	13	13	1.00:1.36	92469
203			Sample = UVB : SMWF + UVB	P14625|ENPL_HUMAN	34.36	13	13	1.00:0.57	92469
204	Nucleosome assembly protein 1-like 1	F8W020	Sample = UVB : SMHF + UVB	tr|F8W020|F8W020_HUMAN	15.22	2	2	1.00:1.35	24366
205	Neutral amino acid transporter B(0)	Q15758	Sample = UVB : SMHF + UVB	Q15758|AAAT_HUMAN	13.69	2	2	1.00:1.38	56598
206			Sample = UVB : SMWF + UVB	Q15758|AAAT_HUMAN	43.27	1	1	1.00:0.48	56598
207	LIM and SH3 domain protein 1 (LASP-1)	A8K1D2	Sample = UVB : SMHF + UVB	tr|A8K1D2|A8K1D2_HUMAN	13.18	1	1	1.00:1.36	29645
208	Polyubiquitin-C	F5GYU3	Sample = UVB : SMEAF + UVB	tr|F5GYU3|F5GYU3_HUMAN	45.46	3	3	1.00:0.56	15032
209			Sample = UVB : SMWF + UVB	tr|F5GYU3|F5GYU3_HUMAN	148.75	3	3	1.00:0.33	15032
210	HNRPCL1 protein	Q6PKD2	Sample = UVB : SMEAF + UVB	tr|Q6PKD2|Q6PKD2_HUMAN	38.05	1	1	1.00:0.46	22652
211	Fascin	B3KTA3	Sample = UVB : SMEAF + UVB	tr|B3KTA3|B3KTA3_HUMAN	29.08	6	6	1.00:0.64	52277
212			Sample = UVB : SMWF + UVB	tr|B3KTA3|B3KTA3_HUMAN	124.82	5	5	1.00:0.36	52277

213	Cathepsin D	A0A1B0GW44	Sample = UVB : SMEAF + UVB	tr|A0A1B0GW44|A0A1B0GW44_HUMAN	20.13	2	2	1.00:0.70	43688
214			Sample = UVB : SMWF + UVB	tr|A0A1B0GW44|A0A1B0GW44_HUMAN	64.61	2	2	1.00:0.49	43688
215	Prothymosin alpha	Q15203	Sample = UVB : SMEAF + UVB	tr|Q15203|Q15203_HUMAN	19.51	1	1	1.00:0.20	8161
216			Sample = UVB : SMWF + UVB	tr|Q15203|Q15203_HUMAN	29.21	1	1	1.00:0.27	8161
217	GTP-binding nuclear protein Ran	P62826	Sample = UVB : SMEAF + UVB	P62826|RAN_HUMAN	17.19	6	6	1.00:0.72	24423
218			Sample = UVB : SMWF + UVB	P62826|RAN_HUMAN	44.52	5	5	1.00:0.41	24423
219	Nucleolar and coiled-body phosphoprotein 1 (NOLC1)	B2RAU8	Sample = UVB : SMEAF + UVB	tr|B2RAU8|B2RAU8_HUMAN	17.76	1	1	1.00:1.39	73604
220	Protein kinase C substrate 80K-H isoform 1	A0A0S2Z4D8	Sample = UVB : SMEAF + UVB	tr|A0A0S2Z4D8|A0A0S2Z4D8_HUMAN	15.01	1	1	1.00:1.35	45124
221	Actin cytoplasmic 1	P60709	Sample = UVB : SMWF + UVB	P60709|ACTB_HUMAN	200	1	1	1.00:0.25	41737
222	Actin cytoplasmic 2	P63261	Sample = UVB : SMWF + UVB	P63261|ACTG_HUMAN	73.56	1	1	1.00:0.47	41793
223	Rab GDP dissociation inhibitor β	P50395	Sample = UVB : SMWF + UVB	P04406|G3P_HUMAN	71.64	13	13	1.00:0.47	36053
224	Eukaryotic translation initiation factor 5A	I3L397	Sample = UVB : SMWF + UVB	tr|I3L397|I3L397_HUMAN	109.85	5	5	1.00:0.39	16019
225	Phosphoglycerate mutase	Q6FHU2	Sample = UVB : SMWF + UVB	tr|Q6FHU2|Q6FHU2_HUMAN	90.08	2	2	1.00:0.43	28804
226	Neutral alpha-glucosidase AB (Epididymis secretory sperm binding protein Li 164nA)	V9HWJ0	Sample = UVB : SMWF + UVB	tr|V9HWJ0|V9HWJ0_HUMAN	87.69	1	1	1.00:0.33	106874

227	Adenosylhomocysteinase	P23526	Sample = UVB : SMWF + UVB	P23526|SAHH_HUMAN	86.64	3	3	1.00:0.44	47716
228	4F2 cell-surface antigen heavy chain	F5GZS6	Sample = UVB : SMWF + UVB	tr|F5GZS6|F5GZS6_HUMAN	85.95	3	3	1.00:0.44	64873
229	Protein SET	A0A0C4DFV9	Sample = UVB : SMWF + UVB	tr|A0A0C4DFV9|A0A0C4DFV9_HUMAN	82.41	3	3	1.00:0.45	31124
230	Inorganic pyrophosphatase (Epididymis secretory sperm binding protein Li 66p)	V9HWB5	Sample = UVB : SMWF + UVB	tr|V9HWB5|V9HWB5_HUMAN	80	2	2	1.00:0.42	32660
231	Aspartate aminotransferase	B3KUZ8	Sample = UVB : SMWF + UVB	tr|B3KUZ8|B3KUZ8_HUMAN	71.52	4	4	1.00:0.47	41326
232	Plasminogen activator inhibitor 2	P05120	Sample = UVB : SMWF + UVB	P05120|PAI2_HUMAN	56.84	4	4	1.00:0.52	46596
233	Acidic leucine-rich nuclear phosphoprotein 32 family member A (ANP32B)	H0YN26	Sample = UVB : SMWF + UVB	tr|H0YN26|H0YN26_HUMAN	56.35	1	1	1.00:0.52	19997
234	Complement component 1 Q subcomponent-binding protein mitochondrial	Q07021	Sample = UVB : SMWF + UVB	Q07021|C1QBP_HUMAN	54.82	3	3	1.00:0.52	31362
235	Fructose-bisphosphate aldolase A	P04075	Sample = UVB : SMWF + UVB	P04075|ALDOA_HUMAN	54.2	5	5	1.00:0.53	39420
236	Transaldolase	A0A140VK56	Sample = UVB : SMWF + UVB	tr|A0A140VK56|A0A140VK56_HUMAN	48.25	3	3	1.00:0.53	37540
237	Macrophage migration inhibitory factor	P14174	Sample = UVB : SMWF + UVB	P14174|MIF_HUMAN	47.72	1	1	1.00:0.55	12476
238	Endoplasmic reticulum resident protein 29	P30040	Sample = UVB : SMWF + UVB	P30040|ERP29_HUMAN	46.09	1	1	1.00:0.49	28993

239	Calmodulin-2	P0DP24	Sample = UVB : SMWF + UVB	P0DP24|CALM2_HUMAN	38.92	2	2	1.00:0.59	16838
240	RAN binding protein 1 isoform CRA_g	A0A140VK94	Sample = UVB : SMWF + UVB	tr|A0A140VK94|A0A140VK94_HUMAN	38.81	2	2	1.00:0.59	23310
241	Vinculin isoform CRA_c	A0A024QZN4	Sample = UVB : SMWF + UVB	tr|A0A024QZN4|A0A024QZN4_HUMAN	36.13	1	1	1.00:0.32	116722
242	Small ubiquitin-related modifier	A0A024R8S3	Sample = UVB : SMWF + UVB	tr|A0A024R8S3|A0A024R8S3_HUMAN	34.8	1	1	1.00:0.37	10871
243	Calnexin	P27824	Sample = UVB : SMWF + UVB	P27824|CALX_HUMAN	34.19	4	4	1.00:0.61	67568
244	Nucleolin	P19338	Sample = UVB : SMWF + UVB	P19338|NUCL_HUMAN	28.76	12	12	1.00:0.49	76615
245	RPLP1 protein	Q6ICQ4	Sample = UVB : SMWF + UVB	tr|Q6ICQ4|Q6ICQ4_HUMAN	24.1	2	2	1.00:0.64	11500
246	FK506 binding protein 12	Q1JUQ3	Sample = UVB : SMWF + UVB	tr|Q1JUQ3|Q1JUQ3_HUMAN	14.33	1	1	1.00:0.12	3965
247	Lysosome-associated membrane glycoprotein 1	B3KRY3	Sample = UVB : SMWF + UVB	tr|B3KRY3|B3KRY3_HUMAN	30.53	2	2	1.00:0.26	42585
248	Transgelin-2	P37802	Sample = UVB : SMWF + UVB	P37802|TAGL2_HUMAN	13.18	9	9	1.00:0.52	22391
